# High-Entropy Electrode Materials: Synthesis, Properties and Outlook

**DOI:** 10.1007/s40820-024-01504-3

**Published:** 2024-09-27

**Authors:** Dongxiao Li, Chang Liu, Shusheng Tao, Jieming Cai, Biao Zhong, Jie Li, Wentao Deng, Hongshuai Hou, Guoqiang Zou, Xiaobo Ji

**Affiliations:** 1 College of Chemistry and Chemical Engineering, Central South University, Changsha, 410083 People’s Republic of China; 2 School of Chemistry and Chemical Engineering, Hunan Institute of Engineering, Xiangtan, 411104 People’s Republic of China

**Keywords:** High-entropy, Energy storage, Electrode materials

## Abstract

High-entropy materials represent a new category of high-performance materials, first proposed in 2004 and extensively investigated by researchers over the past two decades. The definition of high-entropy materials has continuously evolved. In the last ten years, the discovery of an increasing number of high-entropy materials has led to significant advancements in their utilization in energy storage, electrocatalysis, and related domains, accompanied by a rise in techniques for fabricating high-entropy electrode materials. Recently, the research emphasis has shifted from solely improving the performance of high-entropy materials toward exploring their reaction mechanisms and adopting cleaner preparation approaches. However, the current definition of high-entropy materials remains relatively vague, and the preparation method of high-entropy materials is based on the preparation method of single metal/low- or medium-entropy materials. It should be noted that not all methods applicable to single metal/low- or medium-entropy materials can be directly applied to high-entropy materials. In this review, the definition and development of high-entropy materials are briefly reviewed. Subsequently, the classification of high-entropy electrode materials is presented, followed by a discussion of their applications in energy storage and catalysis from the perspective of synthesis methods. Finally, an evaluation of the advantages and disadvantages of various synthesis methods in the production process of different high-entropy materials is provided, along with a proposal for potential future development directions for high-entropy materials.

## Highlights

- The developmental history of high-entropy materials and the conceptual origin of “high entropy” is comprehensively reviewed.
- The preparation methods of various high-entropy electrode materials are comprehensively reviewed.
- The application properties of various high-entropy electrode materials in electrocatalysis and energy storage are comprehensively reviewed, with a prospective outlook on the future development of such materials.

## Graphical abstract

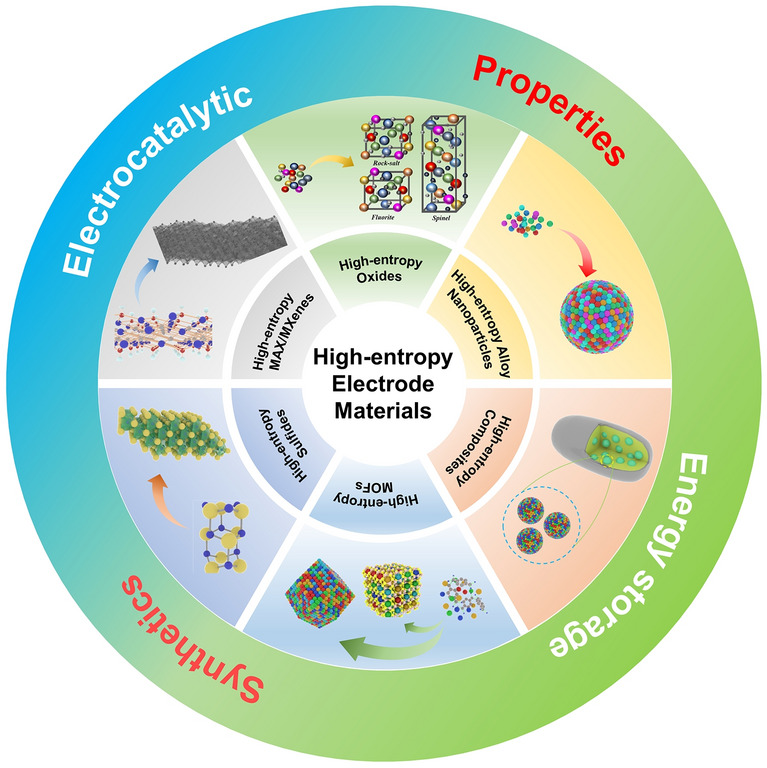

## Introduction

The breakthrough of advanced materials has always been a key factor in changing human society. Materials are closely linked with human development and are essential for the advancement of science and technology. From a historical perspective, in primitive society, the exploration of stone materials marked a fundamental distinction between humans and animals. The use of bronze and iron significantly improved human production capacity, leading to the transition from primitive society to feudal society. In modern times, the Industrial Revolution increased the demand for strong materials. The development of alloy materials such as iron and steel revolutionized large-scale machine production. Additionally, the need for rubber led to the discovery of polymer materials [1]. Alexander Parkes created the first artificial plastic by mixing chloroform and castor oil, resulting in significant changes in people's lifestyles. In the twentieth century, humanity entered an era of continuous exploration into advanced materials. The discovery and application of radioactive elements propelled advancements in nuclear industry development [2]. Organic material developments greatly expanded structural and functional material types [3]. Furthermore, research into superconducting and conductive materials profoundly impacted progress within electronics and energy storage industries [4].

As a new material, high-entropy material (HEM) not only expands the composition space of structural and functional materials, but also introduces new ideas and methods for material design due to its unique entropy effect. This is highly likely to once again revolutionize the way people live in the future [5–7].

Today, with the advancement of human society and advancements in science and technology, there has been extensive exploitation of traditional fossil fuels (coal, oil, natural gas). This not only leads to an energy crisis but also significantly impacts people's lives and health through global warming and pollutant emissions. To address these issues, scientists are dedicated to exploring environmentally friendly renewable clean energy sources [8]. Research has shown that the abundant presence of H_2_O, CO_2_, and N_2_ in the environment can be utilized to produce high-value products such as H_2_, alcohols, and ammonia through electrochemical methods. These materials not only provide direct energy for society but can also be utilized in fuel cells to convert chemical energy into electrical energy for power supply.

Catalytic electrode materials play a crucial role in various electrochemical processes including nitrogen reduction reaction (NRR), carbon dioxide reduction reaction (CO_2_RR), oxygen reduction reaction (ORR), oxygen evolution reaction (OER), hydrogen evolution reaction (HER), and alcohol oxidation reaction (AOR). Among them, alcohol oxidation reactions, redox reactions, and oxygen evolution reactions serve as two-and-a-half reactions for fuel cells and metal-air batteries. Carbon dioxide reduction reactions and NRR help alleviate CO_2_and nitrogen pollution generated during industrial and agricultural production. HERs are essential steps not only in water electrolysis for hydrogen production but also in photoelectrochemical batteries [9, 10], metal-air batteries [11–15], hydrogen fuel cells [16, 17], and hydrolysis batteries [18].

Furthermore, in the utilization of green renewable clean energy sources such as wind power, hydropower, or solar energy, the fluctuating and unstable nature of their power supply necessitates the deployment of increasingly efficient energy storage devices. These include lithium-ion batteries (LIBs), sodium-ion batteries (SIBs), zinc-ion batteries (ZIBs), mixed ion capacitors, and lithium-sulfur batteries among others. Consequently, there is a growing demand for novel potential energy storage electrode materials [19–22].

In recent years, researchers have directed their attention toward electrode materials for energy storage and conversion. Initially, they focused on simple single-metal electrodes and graphite, gradually progressing to alloys, complex carbon materials [23–27], metal–organic frameworks (MOFs) [28–31], organic materials [32], metal oxides [33, 34], sulfides [35, 36], selenides [37], and halides [38–43]. While each of these materials possesses distinct properties individually, their performance alone is no longer sufficient to meet the demands for advanced electrode materials. Consequently, there has been an emergence in the utilization of doping techniques [44–49] as well as composite formation [50–56] and defect design [35, 57–60] for material modification purposes. High-entropy materials incorporating multiple different metal elements can effectively increase entropy by leveraging synergistic effects among metal atoms to alter the crystal lattice structure dynamics and thermodynamics of the material. This alteration ultimately enhances the performance of electrode materials while exhibiting significant potential in advancing electrocatalysis and energy storage technologies [61–65].

However, there are numerous types of high-entropy electrode materials and various preparation methods. Different synthesis methods have distinct effects on material properties. Not all methods can synthesize a variety of elements into a single solid solution phase, and complex preparation methods often limit research on high-entropy electrode materials. Therefore, this paper briefly outlines the development process of high-entropy materials and summarizes the preparation methods and applications of various high-entropy electrode materials, including high-entropy metal (HEM), high-entropy oxide (HEO), high-entropy selenides/sulfide, high-entropy carbides/nitrides, and high-entropy MOF.

## Definition of High-Entropy

"Entropy" is a thermodynamic concept used to measure the regularity of random processes and reveal their uncertainty. The definition of "high-entropy" originally stemmed from the development of high-entropy alloys. In the past, alloys were typically based on a primary component and improved alloy performance by incorporating small amounts of other components. In 1995, the Yeh’s team broke with traditional beliefs by proposing an alloy structure dominated by multiple metallic elements, and in 2004, they first introduced the concept of high-entropy alloys (HEA) [66]. Subsequently, research on high-entropy alloys has experienced explosive growth. High-entropy alloys are a type of super solid solution alloy where solute and solvent elements cannot be distinguished, resulting in the absence of complex structures of intermetallic compounds. They generally consist of five or more metallic or nonmetallic elements in approximately equimolar ratios, with each element composing between 5% to 35% [67]. The ability to form a stable structure is closely related to the Gibbs free energy, as we all know the Gibbs–Helmholtz equation is:

1 $${\Delta G}_{mix}={\Delta H}_{mix}-T{\Delta S}_{mix}$$

According to the Hume-Rothery rules, the incorporation of solute atoms into a multicomponent solid solution will not disturb the crystal structure of the parent phase. Therefore, it is believed by some that the mixing enthalpy of high-entropy alloys, in addition to the atomic size difference and mixing entropy, will also have an impact on the formation of high-entropy alloys. After statistically analyzing the data of mixing enthalpy ΔH_mix_ and atomic size difference δ of a large number of high-entropy alloys [68], the following conclusion is drawn: for disordered solid solutions, − 15 < ΔH_mix_ < 5 kJ mol^−1^, δ < 5%. Further research indicates that analyzing TΔS_mix_/ΔH_mix_ is more meaningful compared to quantitative analysis of high-entropy alloy formation. thus define:

2 $$\Omega ={T}_{m}\Delta {S}_{mix}/\left|\Delta {H}_{mix}\right|$$

where *T*_m_ represents the average melting point of the elements in the alloying group.

High-entropy alloys with simple structures can also be formed without the need for the number of alloying group elements ≥ 5 when Ω > 1 is satisfied.

The concept of "high entropy" can also be defined according to the size of the mixed entropy value, the mixed entropy value ΔS_mix_ is greater than 1.5*R* is called high entropy, according to Boltzmann's formula as well as the additivity of entropy can be obtained by the mixed entropy value of the solid solution alloy [68].

3 $${\Delta S}_{mix}=-R\sum_{i}^{N}{c}_{i}{lnc}_{i}$$

where *R* represents the ideal gas constant, and *c*_i_ denotes the number of moles of component* i*.

**Fig. 1:**
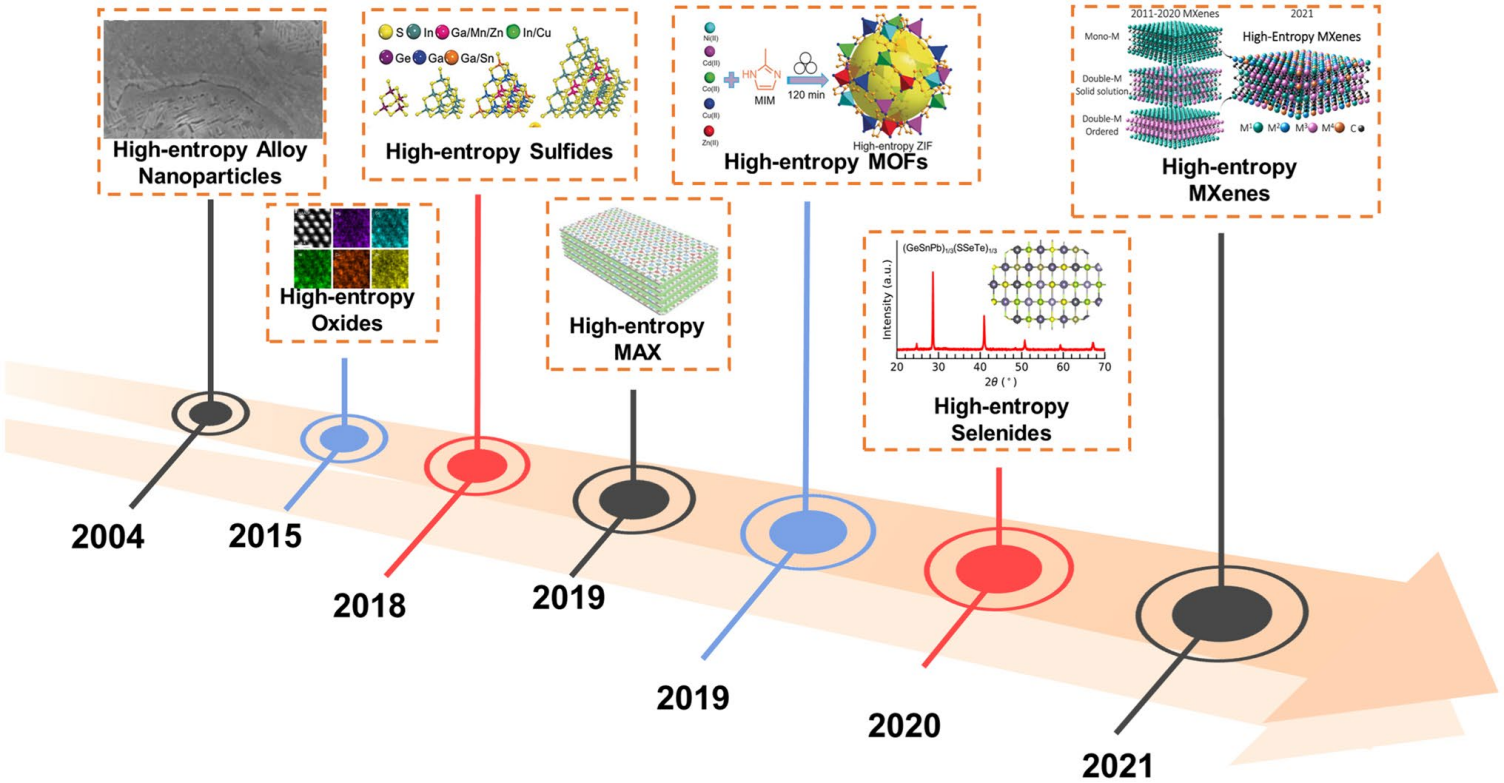
Development of high-entropy electrode materials. Reprinted with permission from Refs. [66, 69, 71–75]. Copyright WILEY‐VCH Verlag GmbH & Co. KGaA, 2015, The Author(s). Published by Springer Nature, 2021, Wiley‐VCH GmbH, 2019, Wiley‐VCH Verlag GmbH & Co. KGaA, 2020, American Chemical Society, 2021, American Chemical Society

In 2015, the concept of high entropy was initially applied to multicomponent oxides. Rost et al. conducted rigorous experiments and developed simple thermodynamic models to demonstrate the significance of entropy in thermodynamics. They also formulated a five-component oxide, further emphasizing the importance of their findings [69]. By increasing the number of components, the system's mixing entropy can be effectively enhanced, leading to the high-entropy effect and the development of high-entropy materials with synergistic effects [70]. As research on high-entropy materials progresses, the definition of high entropy is constantly evolving and improving (Fig. 1). Nowadays, high-entropy materials are mainly categorized into high-entropy alloys, high-entropy oxides, high-entropy sulfides, high-entropy carbides, high-entropy selenides, and high-entropy nitrides etc., all exhibiting a variety of interesting structures and properties.

## High-Entropy Materials

### High-Entropy Alloy

The crystal structure of high-entropy alloys is typically categorized as simple face-centered cubic (FCC), body-centered cubic (BCC), and close-packed hexagonal (HCP) structures. Different atoms randomly occupy lattice sites within the structure, forming a single-phase solid solution structure. Ma et al. suggested that predicting the stability of HEA systems must equally consider the contributions of vibration, electron, and magnetic entropy based on calculations of CoCrFeMnNi high-entropy alloys [110]. Gao et al. investigated the HCP structure of HEA through phase diagram examination, CALPHAD modeling, and molecular dynamics simulations, revealing the significant development potential of single-phase HCP structure HEA containing transition metals and rare earth elements [110]. In high-entropy alloys, no single element exceeds 50% in content to serve as the primary element, thus the characteristics of high-entropy alloys are collectively influenced by all elements. The appropriate elemental composition can be found through Bayesian optimization [111].

Based on the high degree of structural disorder, complexity of elemental composition, and tunability of functionalities in high-entropy materials, Yeh et al. summarized the four major effects of high-entropy alloys from the perspectives of thermodynamics, kinetics, structure, and properties. These effects encompass the thermodynamic high-entropy effect, lattice distortion effect in structure, sluggish diffusion effect in kinetics, and the "cocktail" effect in properties [112]. It is important to note that these four effects serve as a comprehensive summary of the impact that high-entropy materials have in various fields. Furthermore, they are interconnected and interdependent, necessitating a holistic approach.

Since 2004, there has been a growing interest in the remarkable wear resistance, hardness, and corrosion resistance of high-entropy alloys. Consequently, during the first decade following the classification of high-entropy alloys, research predominantly concentrated on understanding the mechanical implications of compositional variations in these alloys and continually enhanced the techniques for their synthesis. The synthesis approaches for high-entropy alloys primarily rely on conventional methods like arc melting and casting. This involves melting various elements using a 500 A current to create a high-entropy alloy, which is then solidified into ingots under 0.01 atm, with homogeneity achievable through repeated cycles [113, 114]. Investigations across different systems have highlighted that the inclusion of Al, V, Ti, Nb, and Mo significantly impacts the hardness and wear resistance of the alloy [115–119]. The effects of varied elemental additions on hardness and wear resistance are often mediated through modifications in the crystal structure. Tong et al. observed that in Al_x_CoCrCuFeNi alloys (*x* = 0–3), a low Al content leads to a simple *fcc* solid solution structure [115]. As the Al content approaches *x* = 0.8, a *bcc* structure emerges, followed by the formation of a mixture of fcc and bcc eutectic phases. An Al content exceeding *x* = 1 results in the generation of modulated plate structures and an organized bcc structure at *x* > 2.8. The alloy's hardness escalates from HV133 to 655 with the increasing Al content, reaching maximum plasticity and hardness at *x* = 0.5. Chenl et al. observed that adding a minute quantity of Ti to Al_0.5_CoCrCuFeNiTi_*x*_ (*x* = 0–2) alloys generates an *fcc* solid solution phase. At *x* = 0.8–1.2, a phase resembling CoCr is formed, and at *x* = 1, a Ti_2_Ni-like phase emerges. The wear resistance exhibits a linear relation with *x* at *x* = 0.6–1, peaks at *x* = 1, and subsequently decreases inversely with *x*. Incorporating Cu, Al, B, and Mo typically influences the corrosion resistance of the alloy [120–123]. The elements' impact on corrosion resistance is usually attributed to their influence on the alloy's passivation ability, thereby affecting its corrosion resistance. Notably, Mo addition often diminishes the metal's corrosion resistance but tends to induce uniform corrosion rather than pitting corrosion, which may impact the internal structure [123]. The addition of Co typically reduces the alloy's compressive strength [124], while Ni addition is linked to crystallization growth resistance and thermal stability [125]. The increasing use of high-entropy alloys has led to a surge in demand for high-entropy alloy thin films. The prevalent technique for fabricating high-entropy alloy thin films is radio frequency sputtering deposition [113, 115, 126, 127], where high-entropy alloys are melted and cast into 5-mm-thick foils as targets, subjected to high-energy particles via a radio frequency sputtering system, followed by depositing sputtered atoms onto a silicon wafer to obtain high-entropy metal thin films. Huang et al. utilized a radio frequency sputtering system to deposit an AlCoCrCu_0.5_NiFe high-entropy alloy oxide film on a silicon wafer. Subsequently, the film was annealed at 500, 700, or 900 °C to obtain an AlCoCrCu_0.5_NiFe high-entropy alloy thin film. When no oxygen was present in the working gas, the film remained amorphous. The introduction of oxygen at levels ranging from 10% to 50% resulted in the formation of an HCP structured oxide film with lattice constants of *a* = 0.3583 nm and *c* = 0.4950 nm. This indicates that varying oxygen content had a significant impact on the structure and properties of the resulting film [126]. During annealing, grain size tended to increase, and intergranular micropores expanded. An et al. performed the preparation of CrCoCuFeNi alloy via a radio frequency magnetron sputtering deposition method, attributing the formation of the solid solution phase to rapid cooling during the sputtering process [127]. Furthermore, other common methods for thin film preparation encompass laser cladding [119], pulsed laser deposition [128], constant potential deposition [129, 130], and detonation spray coating techniques [131]. The study of high-entropy metal thin film preparation methods has significantly advanced the practical developments of high-entropy alloys.

As the exceptional physicochemical properties of high-entropy alloys continue to be investigated, their applications are gradually expanding from structural materials to functional materials.

**Fig. 2:**
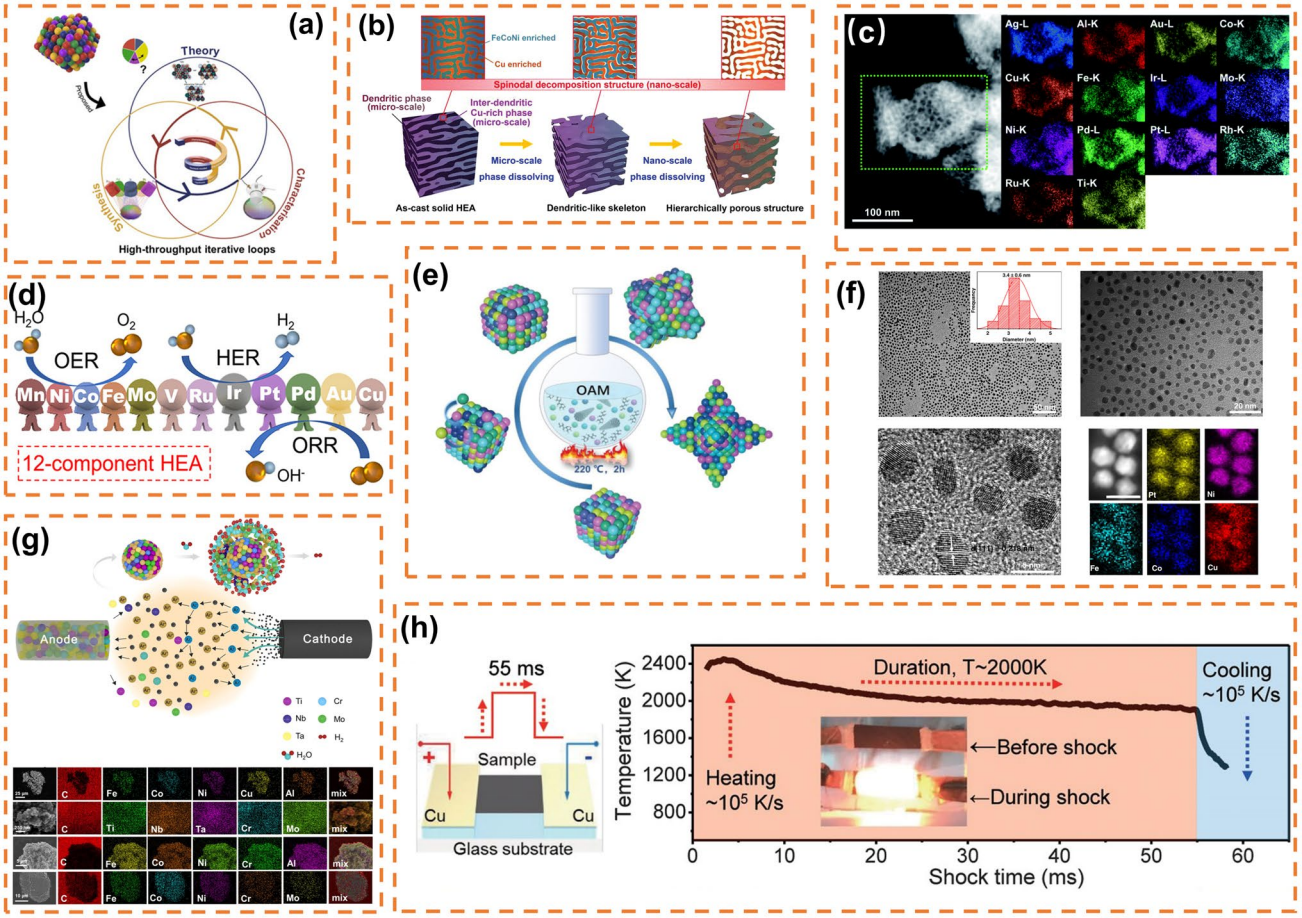
**a** Strategies for validating model hypotheses through computational modeling combined with experimentation. Reprinted with permission from Ref. [133]. Copyright 2021, Angewandte Chemie International Edition published by Wiley–VCH GmbH. **b** Schematic diagram of nanoporous materials constructed by dealloying method. Reprinted with permission from Ref. [82]. Copyright 2022, Advanced Science published by Wiley‐VCH GmbH. **c** High-angle dark field-STEM image of high-entropy alloy with 14 elements by dealloying method. Reprinted with permission from Ref. [78]. Copyright 2021, The Royal Society of Chemistry. **d** Catalytic schematic of high-entropy alloys with 12 elements. Reprinted with permission from Ref. [134]. Copyright 2022, American Chemical Society. **e** Schematic diagram of synthesis of convex cubic high-entropy alloy nanoparticles by low-temperature oil-phase synthesis. Reprinted with permission from Ref. [87]. Copyright 2022, Wiley‐VCH GmbH.** f** TEM image of homogeneous high-entropy alloy synthesized by low-temperature oil-phase method. Reprinted with permission from Ref. [83]. Copyright 2020, The Author(s). Published by Springer Nature. **g** Schematic illustration for the fabrication of high-entropy alloy nanocomposites and their application in seawater splitting and the SEM–EDS of the high-entropy alloy nanocomposites. Reprinted with permission from Ref. [91]. Copyright 2021, Elsevier Inc. **h** Sample preparation and the temporal evolution of temperature during the 55-ms thermal shock. Reprinted with permission from Ref. [93]. Copyright 2018, The American Association for the Advancement of Science

The development of high-entropy alloy nanomaterials is primarily driven by research in the field of electrocatalysis. Since Batchelor et al. in 2019 that high-entropy alloys exhibit nearly continuous adsorption energy distribution and significant catalytic effects on ORR, research on high-entropy alloys in the realm of electrocatalysis has experienced explosive growth [132]. Subsequently, in 2021, Batchelor 's team proposed a novel approach to optimize the model of high-entropy alloys using a method for characterizing high-throughput datasets, thereby determining an unprecedented optimal ratio for solid solution formation during electrocatalytic reactions (Fig. 2a) [133]. The catalytic process typically occurs at the material's surface; thus, a large specific surface area facilitates reactant adsorption. Consequently, the preparation method significantly influences specific surface area and plays a pivotal role in enhancing the electrocatalytic performance of high-entropy alloy nanomaterials (Table 1). Usually, the techniques for producing nanoporous high-entropy alloys primarily encompass the dealloying method [76, 78–80, 82, 134, 135], low-temperature liquid-phase method (also known as solvent thermal method) [84–87], constraint-assisted spark plasma sintering (APS) method [91], and carbon thermal shock method, etc. [94, 136].

**Table 1:** Summary of high-entropy alloys applied to catalysis

HEAs	Synthetic method	Catalytic reaction	Performance	References
AlNiCuPtPdAu-NPs	Dealloying	CO conversion/HER/ORR	Complete conversion at 130 °C / ~ 6.9 A mg^−1^_Pt_ / ∼2.24 A mg^−1^_Pt_	[76]
AlFeCoNiCr	Dealloying	ORR/OER	ORR half-wave potential is ∼0.71 V / η_10_ ∼240 mV (at ∼1.47 V) to reach a current density of 10 mA cm^−2^	[77]
Al_87_Ag_1_Au_1_Co_1_Cu_1_Fe_1_Ir_1_Mo_1_Ni_1_Pd_1_Pt_1_Rh_1_Ru_1_Ti_1_	Dealloying	HER/OER	2.44 A mg^−1^_Pt_ / 274 mV, 121.1 mV dec^−1^ at 10 mA cm^−2^	[78]
Al_88_Ag_1_Au_1_Co_1_Cu_1_Fe_1_Ir_1_Mo_1_Ni_1_Pd_1_Pt_1_Rh_1_Ru_1_	Dealloying	HER/OER	1.70 A mg^−1^_Pt_ / 294 mV, 116.3 mV dec^−1^ at 10 mA cm^−2^	[78]
Al_89_Ag_1_Au_1_Co_1_Cu_1_Fe_1_Ir_1_Ni_1_Pd_1_Pt_1_Rh_1_Ru_1_	Dealloying	HER/OER	1.32 A mg^−1^_Pt_ / 258 mV, 84.2 mV dec^−1^ at 10 mA cm^−2^	[78]
Fe_50_Mn_30_Co_10_Cr_10_	Dealloying	OER	Overpotentials 247, 313 and 362 mV to achieve 10, 50 and 100 mA cm^−2^	[79]
PtPdRhIrNi-NWs	Dealloying	HER	55 mV to drive the current density of 10 mA cm^−2^	[80]
PtPdIrRuAuAg-SNRs	Dealloying	ORR	E_1/2_ = 0.93 V 4.28 A mg _Pt_^−1^ at 0.9 V	[81]
FeCoNiCu	Dealloying	HER	42.2 mV, 31.7 mV dec^−1^ at 10 mA cm^−2^	[82]
Pt_18_Ni_26_Fe_15_Co_14_Cu_27_	Low-temperature oil-phase strategy	HER/MOR	10.96 A mg^−1^_Pt_ at− 0.07 V vs. RHE / 15.04 A mg^−1^_Pt_	[83]
PtRuRhCoNi-NWs	Low-temperature oil-phase strategy	EOR/MOR/HER	9.50 A mg^−1^_Pt_ / 8.20 A mg^−1^_Pt_ / 14.86 A mg^−1^_Pt_ at − 0.05 V_RHE_	[84]
PdFeCoNiCu	Low-temperature oil-phase strategy	HER	6.51 A mg^−1^_Pd_ at − 0.07 V_RHE_	[85]
PtPdRhRuCu MMN	Low-temperature oil-phase strategy	HER	2.7 A mg^−1^_Pt+Pd+Rh+Ru_ at − 0.05 V_RHE_	[86]
Pt_34_Fe_5_Ni_20_Cu_31_Mo_9_	Low-temperature oil-phase strategy	HER/OER/ORR	11.4 A mg^−1^_Pt_ / η_10_ = 259 mV / E_1/2_ = 0.87 V, j_max_ = 5.6 mA cm^−2^ Tafel slope = 69 mV dec^−1^	[87]
PtRhBiSnSb	Low-temperature oil-phase strategy	MOR/EOR/GOR	19.529 A mg^−1^_Pt+Rh_ / 15.558 A mg^−1^ / 7.535 A mg^−1^_Pt+Rh_	[88]
NiCoFePtRh	Low-temperature oil-phase strategy	HER	22.65 mA cm^−2^ at − 0.05 V_RHE_	[89]
PtPdRhRuCu MMN	Low-temperature oil-phase strategy	HER	2.7 A mg^−1^_Pt+Pd+Rh+Ru_ at − 0.05 V_RHE_	[86]
PdPtCuPbBi UNRs	Low-temperature oil-phase strategy	EOR	1.94 A mg^−1^_Pd+Pt_ at 0.45 V_RHE_	[90]
TiNbTaCrMo-NPs	APS	HER	96.33 mV dec^−1^ at 50 mA cm^−2^	[91]
Ru-Rh-Pd-Ir-Pt	APS	HER/ORR/OER	− 0.1, 0.2, and 1.47 V _RHE_	[92]
PtPdRhRuCe	Carbothermal shock	NO_x_ conversion	~ 100% conversion of NH_3_ and > 99% selectivity toward NO_x_ at 700 °C	[93]
Co_0.2_Ru_0.7_Pt_0.1_/PNC NSs	Carbothermal shock	HOR	1.84 A mg^−1^_PGM_ (Pt/C 0.16 A mg^−1^_PGM_)	[94]

#### Dealloying Method

The dealloying method falls within the realm of corrosion engineering. It is a top-down synthesis approach for producing nanoporous high-entropy alloy materials by chemically dissolving specific elements within the synthesized high-entropy alloys (Fig. 2b) [82]. This method is widely utilized due to its simplicity; however, the preparation of high-entropy metal precursors still relies on conventional, energy-intensive methods like melting casting. Qiu et al. employed a method that combines rapid solidification and dealloying to fabricate a multicomponent nanostructured alloy, AlNiCuPtPdAu, termed as nanoporous high-entropy alloy (np-HEA), which exhibits notable high-temperature stability and CO oxidation activity [76]. Fang et al. identified a dual-functional AlFeCoNiCr oxygen electrocatalyst by adjusting the composition of nanostructured HEA/HEO, delivering an open circuit potential of 1.55 V and a high specific capacity of 800 mAh g^−1^ when employed in zinc-air batteries [77]. Cai et al. created a nanoporous ultra-high-entropy alloy consisting of 14 elements through the dealloying method (Fig. 2c) and utilized it in the HER and OER, which also provides a great opportunity for the selection of elements for the synthesis of more catalysts [78]. Yu et al. similarly generated a 12 component nanoporous high-entropy alloy using the dealloying approach (Fig. 2d), demonstrating superior catalytic effects for HER, redox reactions, and OER compared to commercial catalysts and use the np-12 as the cathode of zinc-air battery to verify its excellent performance and stability [134]. Zhou et al. produced a Fe_50_Mn_30_Co_10_Cr_10_ OER electrocatalyst block with excellent catalytic performance using the dealloying technique [79]. Wang et al. synthesized PtPdRhIrNi nanoporous nanowires (NPNWs) by combining rapid solidification and dealloying, introducing a novel family of high-entropy alloys [80]. Li et al. proposed the synthesis of self-supported hierarchical porous high-entropy alloy FeCoNiCu HEA using a physical metallurgy and dealloying strategy, opening the path for developing high-performance porous electrocatalysts by leveraging the chemical and microstructural properties of HEAs [82]. Tao et al. amalgamated multiple metal elements into a single-phase sub-nanometer ribbon, fabricating PtPdIrRuAuAg-SNRs with outstanding electrocatalytic performance, and offering a versatile approach to precisely control the components and concentrations in HEA SNRs [81].

#### Low-Temperature Liquid-Phase Method

The low-temperature liquid-phase reaction method, also known as the one-pot wet chemistry method, involves incorporating pore-forming agents and reducers with the metal precursor into a solvent, followed by stirring and reacting at reduced temperatures to yield high-entropy alloys (Fig. 2e) [87]. For high-entropy alloys, this technique offers the advantage of operating under gentler conditions while enabling precise modulation of individual component concentrations. Li et al. synthesized uniform Pt_18_Ni_26_Fe_15_Co_14_Cu_27_ high-entropy alloy nanoparticles using a straightforward low-temperature oil-phase approach (Fig. 2f) [83]. Their study revealed outstanding electrocatalytic performance of this material for the HER and methanol oxidation reaction (MOR). Additionally, they innovatively developed ultra-thin PtRuRhCoNi high-entropy alloy nanowires (HEA-NWs) with remarkable selectivity for ethanol oxidation reaction (78%), exhibiting superior activity, turnover frequency, and stability for the HER process with PtRuRhCoNi NWs/C [84]. Zhang et al. [85] engineered high-entropy alloy RuFeCoNiCu nanoparticles via a similar low-temperature oil-phase technique, demonstrating its substantial enhancement in the electrocatalytic NRR at lower potentials, resulting in a notable NH_3_ yield (11.4 µg h^−1^ cm^−2^ at 0.05 V). Subsequently, they synthesized PdFeCoNiCu high-entropy alloy, showcasing exceptional catalytic efficiency for the electrocatalytic HER, with Pd and Co identified as the primary active sites for hydrogen generation and water decomposition. Chen et al. [87] fabricated a cubic Pt_34_Fe_5_Ni_20_Cu_31_Mo_9_ high-entropy alloy catalyst using a one-pot method, exhibiting remarkable electrocatalytic performance in the ORR, HER, and OER. Chen et al. reported a one-pot synthesis of hexagonal close-packed (hcp) PtRhBiSnSb high-entropy intermetallic compound (HEI) nanosheets, featuring inherently segregated Pt, Rh, Bi, Sn, and Sb atoms, achieving unprecedented MOR activity under alkaline conditions [88]. Kang et al. employed a one-pot wet chemical reduction method with a diblock copolymer as a soft template to synthesize core–shell-patterned PtPdRhRuCu mesoporous nanospheres (PtPdRhRuCu MMN) [86]. PtPdRhRuCu MMN showcases distinct reduction and growth kinetics from the metal precursor, boasting robust catalytic capability for the HER. Guang Feng and collaborators produced ultra-small NiCoFePtRh high-entropy alloy (us-HEA) nanoparticles with exceptional performance in HER through a versatile and efficient chemical coreduction approach, elucidating comprehensively the atomic, coordination, and electronic structure of us-HEAs [89].

#### Confinement-Assisted Arc and Plasma Shock Method

The confinement-assisted arc and plasma shock (APS) method employs constrained assistive APS to generate a high-energy plasma arc for bombarding multimetal powders, leading to the production of gaseous metal atoms and the formation of nanoscale multimetallic alloys during rapid cooling (Fig. 2g). Wang et al. [91] synthesized TiNbTaCrMo HEA-NPs using this approach and identified its potential as a viable candidate for the electrocatalytic HER in natural seawater. Banko et al. [92] combined co-sputtering with shadow masking to create multiple microscale composite libraries in a deposition process. Additionally, Yoshihiro Chida [137] proposed an experimental research platform for synthesizing atomically controlled single-crystal high-entropy alloy surfaces in a vacuum and prepared nanothick Pt and equiatomic ratio Cr–Mn–Fe–Co–Co-Ni epitaxial layers on a Pt substrate, evaluating their catalytic activity. Li et al. [90] introduced a programmable method for manufacturing nanoscale HEAs with controllable composition and structure, enabling the combination of five or more elements such as Pd/Pt/Ag/Cu/Fe/Co/Ni/Pb/Bi/Sn/Sb/Ge. They successfully produced PdPtCuPbBi UNRs using a two-step template-directed synthesis.

#### Carbon Thermal Shock Method

The carbon thermal shock method is employed to synthesize nanoparticles with desired chemical compositions, sizes, and phases by loading a mixture of precursor metal salts onto a carbon carrier and subjecting it to continuous thermal shocks at approximately 2000 K. Yao et al. [93] have pioneered the development of a carbon thermal shock platform suitable for high-entropy alloying (Fig. 2h), enabling the preparation of high-entropy alloying nanoparticles containing eight different metal elements. Furthermore, the synthesis of high-entropy alloying nanoparticles using the carbon thermal shock method is not limited to mixtures of metal salts alone. Additionally, Qiu et al. [94] have successfully synthesized Co_0.2_Ru_0.7_Pt_0.1_/PNC NSs through a general 2D MOF-assisted pyrolysis-replacement-alloying route, suggesting this as an appealing synthetic approach for constructing high-performance multimetal nanomaterials. Notably, Cha et al. [94] in recent years, reported a rapid flash thermal shock method for synthesizing high-entropy nanoparticles on carbon nanofiber carriers with significantly enhanced synthesis rates.

In conclusion, high-entropy alloy materials form the foundation of high-entropy material research and serve as the defining elements of high-entropy materials. High-entropy alloy bulk materials, thin film materials (including high-entropy ceramics and glass), are widely applied as structural materials. Researchers have conducted extensive studies on the impact of different metal elements and element ratios on their structure. In terms of electrocatalysis, high-entropy alloy nanomaterials (such as nanoparticles and nanowires) play a crucial role. The precursors for dealloying process need to be synthesized using traditional alloy synthesis methods, leading to drawbacks such as high energy demand, stringent equipment requirements, and a two-step synthesis process to obtain the material. The carbon thermal shock method is capable of easily synthesizing high-entropy alloy nanomaterials with multiple metal elements. However, due to the high temperature required, it has significant energy and equipment requirements, making it uneconomical for synthesis and limiting the final product shape and size. Nevertheless, its rapid synthesis and simple steps give it potential for large-scale preparation. The confinement-assisted arc and plasma shock (APS) method show great potential in research due to their high precision in controlling the incorporation of metal elements at the atomic level. However, they have the highest equipment requirements and costs and do not have potential for large-scale preparation. On the other hand, the low-temperature liquid-phase synthesis method offers mild conditions and lower economic costs. It allows for easy adjustment of metal crystal structure and can synthesize microstructures more suitable for electrocatalysis. Nonetheless, this method faces a higher synthesis energy barrier for high-entropy alloys compared to medium–low entropy alloys due to its limited energy provision. Additionally, it requires thorough mixing of components and has a limited diffusion rate in the liquid phase, making it unsuitable for large-scale synthesis. Therefore, the APS method and low-temperature liquid-phase method are expected to undergo long-term development in experimental findings. Despite the economic disadvantages of the carbon thermal shock method, it remains the preferred choice for industrialization.

### High-Entropy Oxides

Since the groundbreaking work by Rost et al. in 2015 [69], which demonstrated the synthesis of high-entropy solid solution oxides, the application of these materials has progressively extended beyond structural applications to encompass electrochemical energy storage systems [138]. Xu et al. [139] have demonstrated that high entropy can enhance the protonation ability of the oxide and facilitate the movement of the O-p band center toward the Fermi level. This, in turn, leads to an improvement in the performance of the oxide as a cathode material for batteries. High-entropy oxides are characterized by their incorporation of multiple metallic elements (typically more than five) and exhibit diverse crystal structures, including perovskite, rock salt, and spinel structures. During the process of charging and discharging, the crystal structure of the oxide will undergo changes in response to variations in voltage. The key to enhancing its cycle stability lies in designing a more reversible high-entropy oxide material. The choice of crystal structure significantly influences the performance and utilization potential of high-entropy oxides. At the same time, the variation in elements also significantly impacts the characteristics of high-entropy oxides. When all metal elements are transition metals, they typically exhibit a substantial dielectric constant. Integration of rare earth elements results in high-entropy rare earth oxides with a narrower band gap [140]. Furthermore, when both transition metal and rare earth elements are integrated, they demonstrate even greater potential characteristics (Table 2).

**Table 2:** Summary of high-entropy oxides applied to energy storage

HEOs	Method	Structure	Battery	Electrode	Performance	References
(MnFeCoNiCr)_3_O_4_	Surfactant-assisted hydrothermal	Spinel	LIBs	Anode	1235 mAh g^−1^ (500 mA g^−1^)	[95]
(FeCoNiCrMn)_3_O_4_	High-temperature solid-state reaction	Spinel	LIBs	Anode	Discharge and charge1034/680 mAh g^−1^ (500 mA g^−1^ first cycle)	[96]
(Al_0.2_CoCrFeMnNi)_0.58_O_4_	High-temperature solid-state reaction	Spinel	LIBs	Anode	554 mAh g ^−1^ (200 mA g^−1^ after 500 cycle)	[97]
(FeCoNiCrMnCuLi)_3_O_4_	High-temperature solid-state reaction	Spinel	LIBs	Anode	Discharge and charge1000.1/626.6 mAh g^−1^ (50 mA g^−1^ first cycle)	[98]
(FeCoNiCrMnMgLi)_3_O_4_	High-temperature solid-state reaction	Spinel	LIBs	Anode	discharge and charge1031/592.1mAh g^−1^ (50 mA g^−1^ first cycle)	[98]
(FeCoNiCrMnZnLi)_3_O_4_	High-temperature solid-state reaction	Spinel	LIBs	Anode	Discharge and charge1049.9/706 mAh g^−1^ (50 mA g^−1^ first cycle)	[98]
(FeNiCrMnMgAl)_3_O_4_	Solution combustion synthesis	Spinel	LIBs	Anode	657 mAh g^−1^ (200 mA g^−1^ after 200 cycle)	[99]
(CrFeMnNiCo_2_)_3_O_4_	Solution combustion synthesis	Spinel	LIBs	Anode	Stable capacity 467.8 mAh g^−1^ (200 mA g^−1^)	[100]
(CrFeMnNiCo_3_)_3_O_4_	Solution combustion synthesis	Spinel	LIBs	Anode	stable capacity 574.1 mAh g^−1^ (200 mA g^−1^)	[100]
(CrFeMnNiCo_4_)_3_O_4_	Solution combustion synthesis	Spinel	LIBs	Anode	Stable capacity 506.2 mAh g^−1^ (200 mA g^−1^)	[100]
(CrFeMnCoMgLi)_3_O_4_	Solution combustion synthesis	Spinel	LIBs	Anode	Stable capacity 393.1 mAh g^−1^(100 mA g^−1^)	[101]
(CrFeMnCoMg)_3_O_4_	Solution combustion synthesis	Spinel	LIBs	Anode	Stable capacity 395.3 mAhg^−1^ (100 mA g^−1^)	[101]
(CrFeMnCoNiLi)_3_O_4_	Solution combustion synthesis	Spinel	LIBs	Anode	stable capacity 395 mAh g^−1^ (100 mA g^−1^)	[101]
(CrFeMnCoNi)_3_O_4_	Solution combustion synthesis	Spinel	LIBs	Anode	Stable capacity 407.8 mAh g^−1^ (100 mA g^−1^)	[101]
(MgTiZnNiFe)_3_O_4_	Solid-state sintering	Spinel	LIBs	Anode	Discharge and charge 166.8/424.7 mAh g^−1^ (100 mA g ^−1^ first cycle)	[102]
(CoTiZnNiFe)_3_O_4_	Solid-state sintering	Spinel	LIBs	Anode	Discharge and charge 423.9/674.7mAh g ^−1^ (100 mA g^−1^ first cycle)	[102]
(Cr_0.2_Mn_0.2_Fe_0.2_Co_0.2_Ni_0.2_)_3_O_4_	Solid-state reaction	Spinel	LIBs	Anode	Stable capacity 560 mAh g^−1^ (100 mA g^−1^)	[103]
(FeCoNiCrMn)_3_O_4_	Solid-state reaction	Spinel	LIBs	Anode	692.8 mAh g ^−1^ (500 mA g^−1^ after 260 cycle)	[104]
[(Bi,Na)_1/5_(La,Li)_1/5_(Ce,K)_1/5_Ca_1/5_Sr_1/5_]TiO_3_	Solid-state reaction	Perovskite	LIBs	Anode	120.4 mAh g ^−1^ (1000 mA g^−1^ after 300 cycle)	[105]
Na_0.7_Mn_0.4_Ni_0.3_Cu_0.1_Fe_0.1_Ti_0.1_O_1.95_F_0.1_	Solid-state reaction	O3/P2-tpye	SIBs	Cathode	86.7 mAh g^−1^ (800 mA g^−1^)	[106]
NaNi_0.12_Cu_0.12_Mg_0.12_Fe_0.15_Co_0.15_Mn_0.1_Ti_0.1_Sn_0.1_Sb_0.04_O_2_	Solid-state reaction	O3-type	SIBs	Cathode	Stable capacity 110 mAh g^−1^ (12 mA g^−1^)	[107]
(MgCoNiCuZn)O	Nebulized spray pyrolysis	Rock-salt	LIBs	Anode	Stable capacity 549 mAh g^−1^ (100 mA g^−1^)	[108]
(LiMgCoNiCuZn)O	Nebulized spray pyrolysis	Rock-salt	LIBs	Anode	Stable capacity 714 mAh g^−1^ (100 mA g^−1^)	[108]
(Co_0.2_Cr_0.2_Fe_0.2_Mn_0.2_Ni_0.2_)_3_O_4_	Solgel method	Spinel	SCs	Cathode	75 F g^−1^ (1 A g^−1^)	[109]

**Fig. 3:**
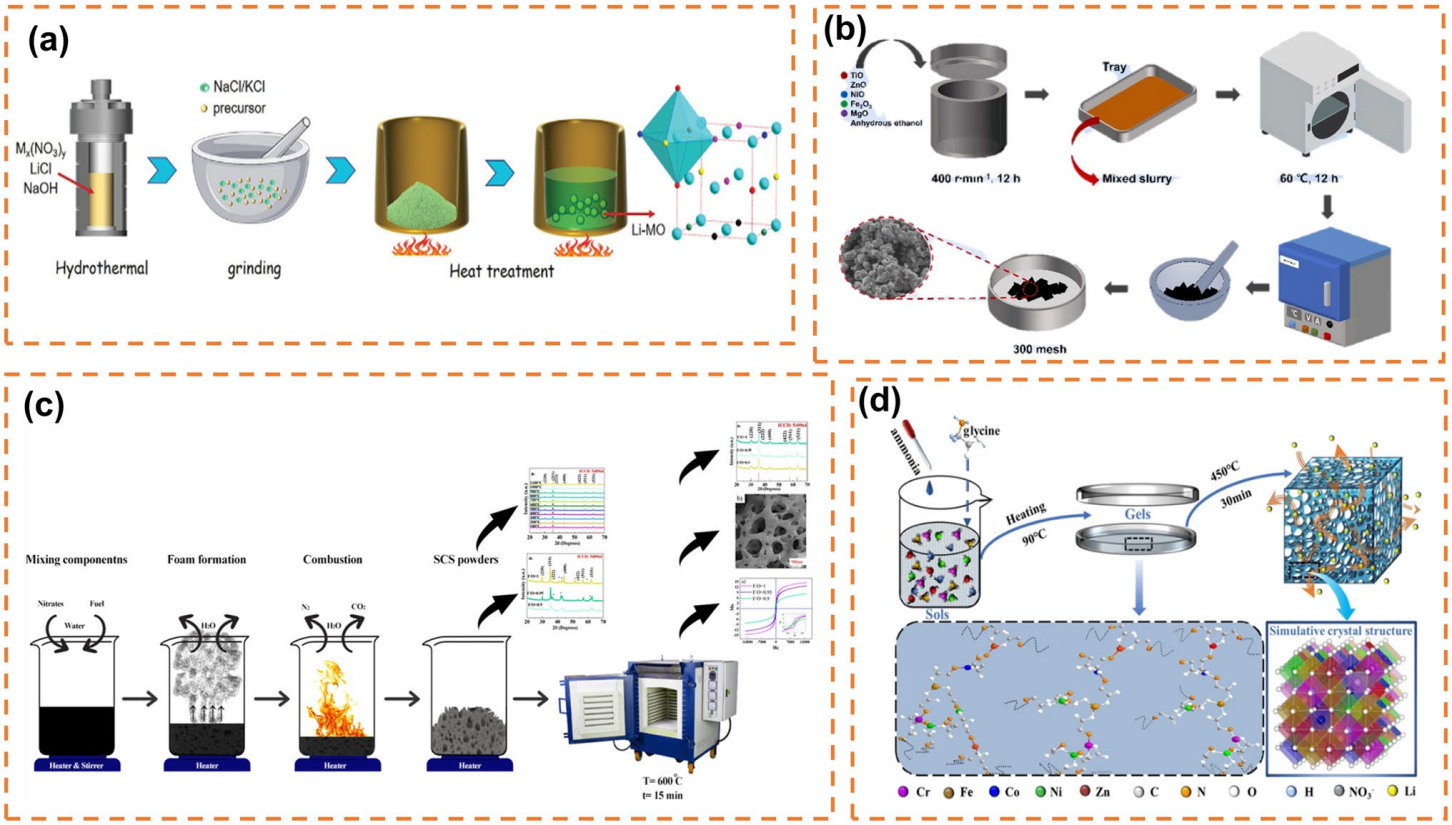
**a** Schematic diagram of the preparation procedure. Reprinted with permission from Ref. [108]. Copyright 2022, Wiley‐VCH GmbH.** b** Schematic diagram illustrating the preparation of high-entropy oxide electrode materials. Reprinted with permission from Ref. [102]. Copyright 2023, Elsevier Ltd. **c** Schematic of synthesis (Co, Cr, Fe, Mn, Ni)_3_O_4_ HEO through the SCS method. Reprinted with permission from Ref. [141]. Copyright 2023, Elsevier Inc. **d** Schematic illustration of the synthesis process and crystal structure of HEO. Reprinted with permission from Ref. [142]. Copyright 2022, American Chemical Society

The synthesis methods of high-entropy oxides mainly include solvothermal method (Fig. 3a) [95, 108], solid-phase reaction method (Fig. 3b) [102], solution combustion synthesis method (Fig. 3c) [141], gel method and other synthesis methods (Fig. 3d) [142].

#### Solvothermal Method

**Fig. 4:**
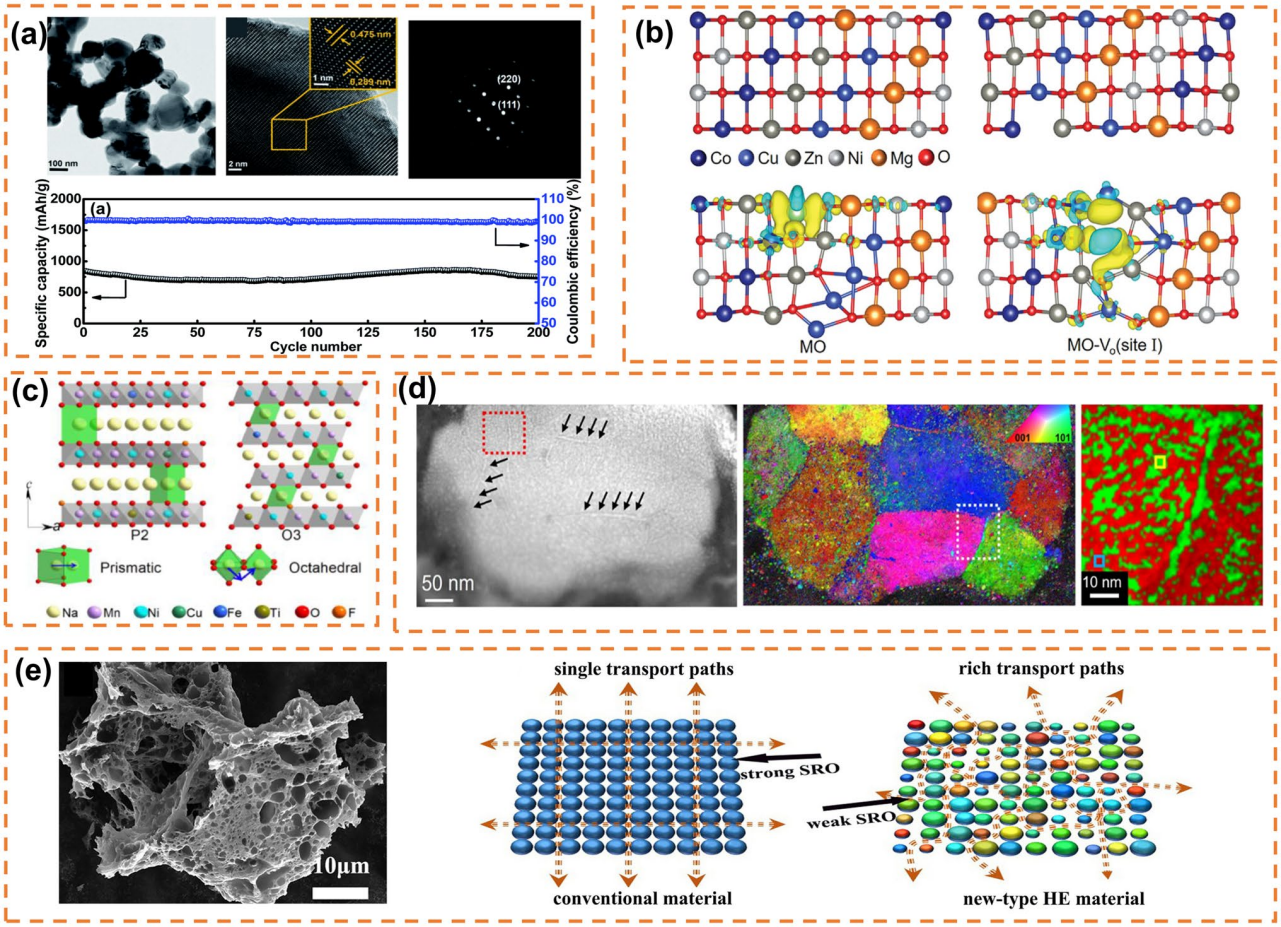
**a** TEM, HRTEM, SAED data of HEO NPs prepared by hydrothermal method and their cycling stability. Reprinted with permission from Ref. [95]. Copyright 2020, The Royal Society of Chemistry. **b** Atomic structure models of Li-HEO and Li-HEO-Vo as well as the charge density difference of Li in HEO and Li-HEO-Vo. Reprinted with permission from Ref. [108]. Copyright 2022, Wiley‐VCH GmbH. **c** Schematic crystalline structure of P2 and O3 phase. Reprinted with permission from Ref. [106]. Copyright 2023, Elsevier B.V. **d** HAADF-STEM image as well as the orientation map obtained and typical phase map corresponding by indexing the diffraction patterns of the 4D-STEM data. Reprinted with permission from Ref. [148]. Copyright 2023, The Author(s). Published by Springer Nature. **e** SEM image of HEO powder prepared by gel method and the schematic diagram of ion pathways in conventional and high-entropy materials. Reprinted with permission from Ref. [142]. Copyright 2022, American Chemical Society

The solvent thermal method of oxides is to obtain oxides by dissolving metal salts in solvents, adding precipitant and reacting in a hydrothermal kettle. High-performance lithium-ion batteries often utilize high-entropy oxide spinel structure materials. Nguyen et al. synthesized (MnFeCoNiCr)_3_O_4_ particles through a surfactant-assisted hydrothermal method (Fig. 4a). After 200 cycles at a charge–discharge rate of 500 mA g^−1^, the particles exhibited a capacity retention rate of 90% (1235 mAh g^−1^) and demonstrated excellent rate performance (500 mAh g^−1^ at 2000 mA g^−1^) [95]. Similarly, high-entropy oxide rock salt materials with a similar structure to spinel are extensively employed in lithium-ion batteries. Cheng et al. investigated the lattice distortion of high-entropy oxides under pressure by studying the rock salt phase (Co_0.2_Cu_0.2_Mg_0.2_Ni_0.2_Zn_0.2_)O, revealing its highly adjustable nature [143]. Liu et al. [108] using the hydrothermal method (Fig. 4b), introduced ions with targeted functions into oxygen vacancies of rock salt (MgCoNiCuZn)O, thereby enhancing ion/electron transmission kinetics and achieving stable discharge capacity of MO at 549 mAh g^−1^ under a current density of 0.1 A g^−1^; moreover, Li-MO exhibited an increased specific capacity up to 714 mAh g^−1^, highlighting the potential for structural and compositional adjustability as well as promising development prospects offered by rock salt oxides. In October 2022, Biesuz et al. [144] employing manganese instead of nickel through hydrothermal synthesis, developed the first nickel-free high-entropy rock salt material (Mg, Co, Mn Cu Zn)O that improved safety and environmental friendliness while maintaining conductivity higher than 10^–3^ S cm^−1^ and stability characteristics even at elevated temperature such as 80 °C. Su et al. [145] elucidated the composition-dependent transformation/alloying reaction kinetics and the spatiotemporal changes in valence state during lithiumization by investigating the diverse reaction kinetics and structural evolution of rock salt HEO throughout cycling, thereby offering valuable insights for the design of enhanced lithium storage devices. He et al. [146] synthesized (CoCuFeMnNi)_3_O_4_ using a microwave-assisted solvothermal method, which significantly reduces the time required for solvothermal synthesis and allows for precise control at the nanoparticle scale.

#### *Solid-Phase* Reaction Method

The solid-phase preparation method for high-entropy oxides involves obtaining the material by calcining the precursor in an oxygen atmosphere. Wang et al. successfully synthesized a single-phase spinel structure (FeCoNiCrMn)_3_O_4_ through high-temperature solid-state reaction at 900 °C. This material exhibited a high specific capacity of 1034/680 mAh g^−1^ (discharge/charge) and excellent rate performance of 182 mAh g^−1^ at 2 A g^−1^. In situ high-temperature X-ray diffraction was employed to investigate the structural evolution with increasing calcination temperature [96]. Duan et al. prepared spinel structure oxides of 7 metal elements (FeCoNiCrMnXLi)_3_O_4_ (*X *= Cu, Mg, Zn) by solid-state reaction, in which the (FeCoNiCrMnZnLi)_3_O_4_ negative electrode exhibited better electrochemical lithium storage performance in the three samples with discharge specific capacities of 695, 577, 460, 336, 250, and 173 mAh g^−1^ at 50, 100, 200, 500, 1000, and 2000 mA g^−1^ respectively. Sun et al. prepared high-entropy (Cr_0.2_Mn_0.2_Fe_0.2_Co_0.2_Ni_0.2_)_3_O_4_ with *Fd3m* spinel structure by solid-state reaction, with a high specific capacity of 560 mAh g^−1^ at 100 mA g^−1^ and excellent capacity retention of 100% after 5000 cycles [103]. High-entropy perovskite oxide crystalline materials have been widely studied for their use as cathode materials in fuel cells, reversible proton ceramic electrochemical batteries, and electrocatalysts in lithium-sulfur batteries. Yan et al. synthesized high-entropy perovskite oxide (HEPO) [(Bi, Na)_1/5_(La, Li)_1/5_(Ce, K)_1/5_Ca_1/5_Sr_1/5_]TiO_3_ as a negative electrode material for lithium-ion batteries by solid-phase reaction method, and obtained 120.4 mAh g^−1^ reversible capacity and nearly 100% capacity retention rate at 1 A g^−1^ current density after 300 cycles. O3-type layered high-entropy oxides can effectively inhibit the intermediate phase transition in the electrochemical reaction process, inhibit the order of charge and sodium vacancy, and thus inhibit the interlayer sliding and phase transition defects of layered structure electrodes [105]. Zhou prepared P2/O3 biphasic high-entropy oxide Na_0.7_Mn_0.4_Ni_0.3_Cu_0.1_Fe_0.1_Ti_0.1_O_1.95_F_0.1_ (Fig. 4c) by solid-phase method and found that it has excellent capacity retention in a wide temperature range (− 40 to 50 °C) [106]. ChanQin Duan designed and prepared a new six-component high-entropy oxide (HEO) layered cathode Na(Fe_0.2_Co_0.2_Ni_0.2_Ti_0.2_Sn_0.1_Li_0.1_)O_2_ by high-temperature solid-phase method [98]. Wu et al. conducted the synthesis of high-entropy rock salt oxides, high-entropy spinel oxides, and high-entropy perovskite oxides using the rapid Joule thermal synthesis method, which involves burning nickel foil. They also demonstrated the OER activities of these synthesized materials [147].

#### Solution Combustion Synthesis Method

Solution combustion synthesis (SCS) is essentially an intense exothermic process, which is achieved by dissolving the metal salts in deionized water, heating to about 330 °C, adding aluminum foil and various fuels (glycine, urea, and hexaamine, etc.) and waiting for the system to continue exothermic combustion to obtain samples [141]. Xiang et al. [97] synthesized spinel-type (Al_0.2_CoCrFeMnNi)_0.58_O_4-δ_ HEO nanocrystalline powders with high concentration of oxygen vacancies by solution combustion synthesis method. Compared with (CoCrFeMnNi)_0.6_O_4-δ_, the inactive Al^3+^-doped (Al_0.2_CoCrFeMnNi)_0.58_O_4_-δ negative electrode provided a reversible specific capacity of 554 mAh g^−1^ after 500 cycles at a specific current of 200 mA g^−1^ more than twice that of the undoped, accompanied by good rate performance (634 mAh g^−1^ even at 3 A g^−1^) and cycling performance. Zheng et al. [99] prepared (FeNiCrMnMgAl)_3_O_4_ spinel high-entropy oxides by solution combustion synthesis method and ball milling refining process for lithium-ion batteries, which had a capacity of 657 mAh g^−1^ after 200 cycles at a current density of 0.2 A g^−1^ and also had good rate performance (350 mAh g^−1^ at 4 A g^−1^). Liu et al. synthesized three groups of high-entropy spinel oxides (CrFeMnNiCo_*x*_)_3_O_4_ (*x* = 2,3,4) by solution combustion method, with reversible capacities of 467.8, 574.1, and 506.2 mAh g^−1^ at 200 mA g^−1^ respectively, and four new spinel high-entropy oxides by glycine-nitrate solution combustion method [100, 101]. Su et al. prepared (MgTiZnNiFe)_3_O_4_ and (CoTiZnNiFe)_3_O_4_ by solid-state combustion method and verified the lithium storage mechanism of the materials by in situ ED characterization [102]. Wang et al. explained that the "cocktail effect" was due to more cations which could cause the oxide to self-assemble into micron-scale particles (Fig. 4d) without nanoscale pre-modification of the metal oxides by analyzing the electrochemical reaction of Mg_0.2_Co_0.2_Ni_0.2_Cu_0.2_Zn_0.2_, indicating that element diversity is the key to optimize the cationic electrode materials [148]. Xiao et al. successfully prepared (FeCoNiCrMn)_3_O_4_ by oxidizing FeCoNiCrMn alloy powder and proposed that high entropy makes the oxide have a stable structure and narrow band gap, and spinel structure provides a channel for ion transport through the study of (FeCoNiCrMn)_3_O_4_ [104].

#### Solgel Method

The solgel method, similar to the hydrothermal method, involves dissolving a transition metal salt in deionized water and adding it to a solution of acrylamide, N–N dimethyl diacrylamide, and ammonium persulfate. The mixture is vigorously stirred to form a wet gel which is subsequently dried, calcined, and ground under mild reaction conditions to obtain high-entropy oxide powder. Li et al. successfully synthesized (Co_0.2_Cr_0.2_Fe_0.2_Mn_0.2_Ni_0.2_)_3_O_4_ using the polyacrylamide gel method and observed its excellent electrochemical performance in supercapacitors. Yang et al. synthesized porous spinel structure high-entropy oxide (Cr_0.2_Fe_0.2_Co_0.2_Ni_0.2_Zn_0.2_)_3_O_4_ via the solgel method at low temperature, demonstrating a remarkable specific capacity of 1022 mAh g^−1^ after 1000 cycles at 1 A g^−1^ as an anode material for lithium-ion batteries. The authors attributed this exceptional capacity performance to the nanostructure generated through the solgel method, which effectively suppressed volume expansion and altered ion spacing due to lattice distortion caused by different metal ions in high-entropy materials, thereby facilitating enhanced ion transport pathways (Fig. 4e) [142].

In conclusion, high-entropy oxides have garnered significant attention due to their high electrical conductivity, large dielectric constant, narrow bandgap, and ease of creating vacancies. These characteristics make them promising candidates for high-performance battery electrode materials and demonstrate good performance in electrocatalytic fields such as OER and HER.The solvothermal method is a widely used synthesis method for high-entropy oxides. It is simple and mild but typically requires insulation for more than 10 h to allow for crystal growth. The provided energy is limited, and the insulation temperature needs to be adjusted according to the metal elements. Some researchers also utilize microwave technology.The solid-phase reaction method includes the high-temperature solid-phase method, high-energy ball milling method, and joule heating method. While the ball milling synthesis method is simple, it cannot guarantee the uniformity of the formed nanoparticles. The high-temperature solid-phase method and joule heating method are characterized by high energy consumption. Additionally, the process of quenching in air may lead to the formation of cracks due to structural transformation, thereby posing challenges in achieving a single-phase structure. The synthesis conditions of the solgel method are comparatively gentler than those of other methods. With the presence of organic template molecules, this approach allows for precise control over pore size and other nanostructures, resulting in a more uniform nanoparticle synthesis. Furthermore, its low temperature ensures that the structure remains undamaged, which is advantageous for catalysis and energy storage performance. However, the limited energy input provided during the synthesis process using solgel methods makes it challenging to synthesize complex high-entropy materials, thereby restricting its application within this field.

### High-Entropy Sulfides/Selenides

**Fig. 5:**
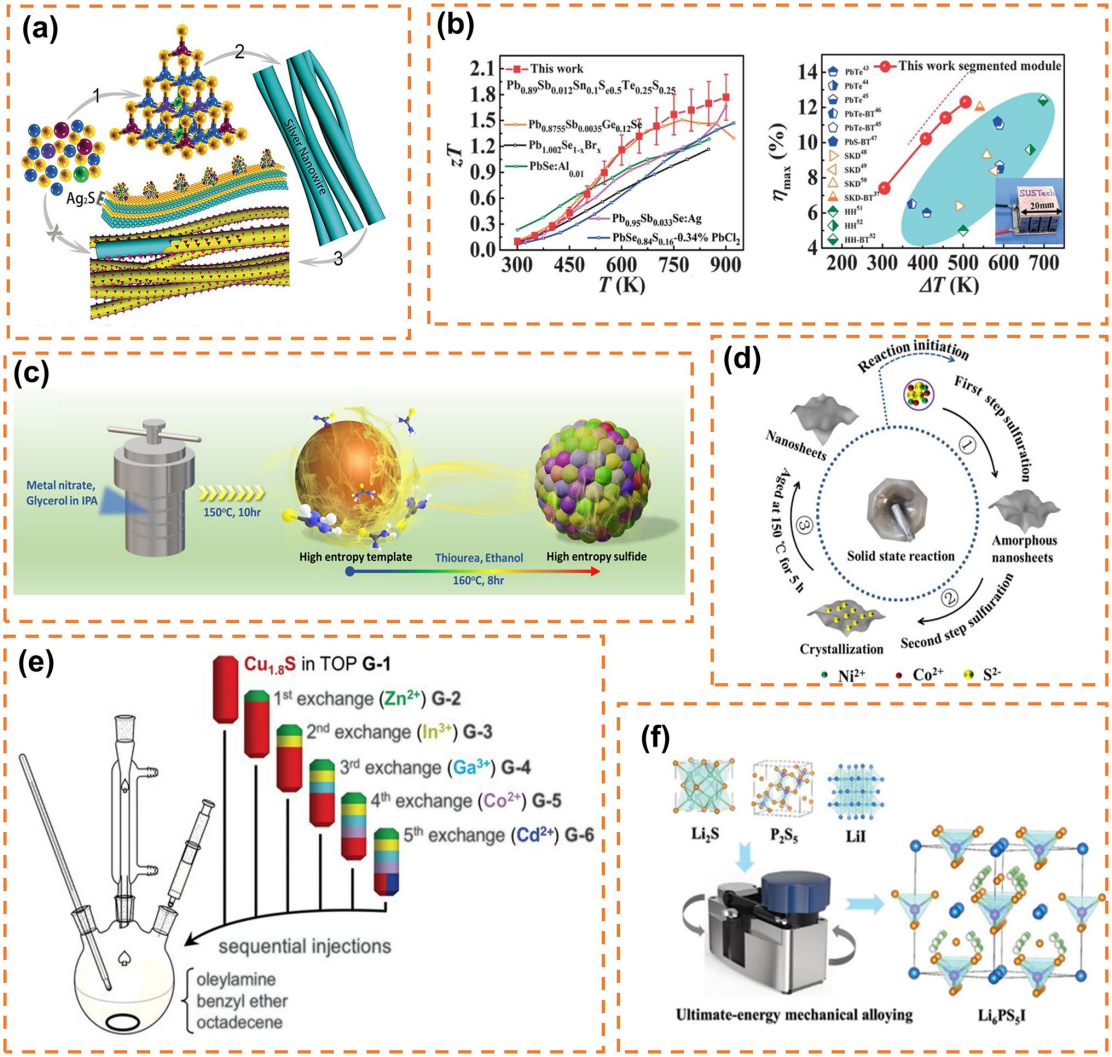
**a** Schematic diagram of nano silver wire/sulfide heterostructure prepared by simple etching and growth method. Reprinted with permission from Ref. [71]. Copyright 2018, Wiley‐VCH Verlag GmbH & Co. KGaA. **b** Change of zT value of high entropy PbSe based material with temperature and the maximum conversion efficiency (hmax) of a high-entropy segmented thermoelectric module varies with temperature difference (DT). Reprinted with permission from Ref. [150]. Copyright 2021, The American Association for the Advancement of Science. **c** Schematic diagram of synthesis of high-entropy sulfide by solvothermal method. Reprinted with permission from Ref. [152]. Copyright 2023, Elsevier B.V. **d** Preparation of Co doped Ni_3_S_4_ by solid-state reaction method. Reprinted with permission from Ref. [153]. Copyright 2019, Elsevier B.V. **e** Schematic diagram of preparation of high-entropy sulfide by cation exchange method. Reprinted with permission from Ref. [154]. Copyright 2020, The American Association for the Advancement of Science. **f** Schematic diagram of the preparation of Li_6_PS_5_I electrolyte using UEMA method. Reprinted with permission from Ref. [156]. Copyright 2021, Wiley‐VCH GmbH

Due to the existence of different growth and reaction rates leading to phase separation, M-S bond length mismatch and other problems limiting the development of traditional polymetallic sulfides, people have studied high-entropic sulfides later than oxides. In 2018, Liu et al. proposed for the first time a method of integrating polymetallic sulfide clusters on silver nanowires and preparing multicomponent metal by a simple etch growth sulfide heterostructures strategy (Fig. 5a), which provides a new synthetic idea for high-entropy metal sulfides in the future. High-entropy metal sulfides were firstly used to study thermoelectric materials [149, 150] and high-entropy selenides and tellurides, which also belong to the sulfur group of compounds, were firstly developed in the field of thermoelectricity [151], and only in recent years have there been reports in the literature in the field of energy storage. Jiang et al. through the study of entropy-driven structurally stabil0 formation of n-type PbSe-based high-entropy materials, introduced different kinds of atoms into PbSe to carry out the configurational entropy modulation, it was found that the large strain generated by the severely distorted lattice in the high-entropy material provides strong scattering of heat-carrying phonons, resulting in an ultra-low lattice thermal conductivity (κL). High-quality factor (zT) and conversion efficiency (η) of high-entropy materials and modules are thus achieved (Fig. 5b). The main methods for the preparation of high-entropy sulfides are solvothermal (Fig. 5c) [152], solid-state reaction synthesis (Fig. 5d) [153], cation-exchange (Fig. 5e) [154], and mechanical alloying (Fig. 5f) [155, 156].

#### Solvothermal Method

**Fig. 6:**
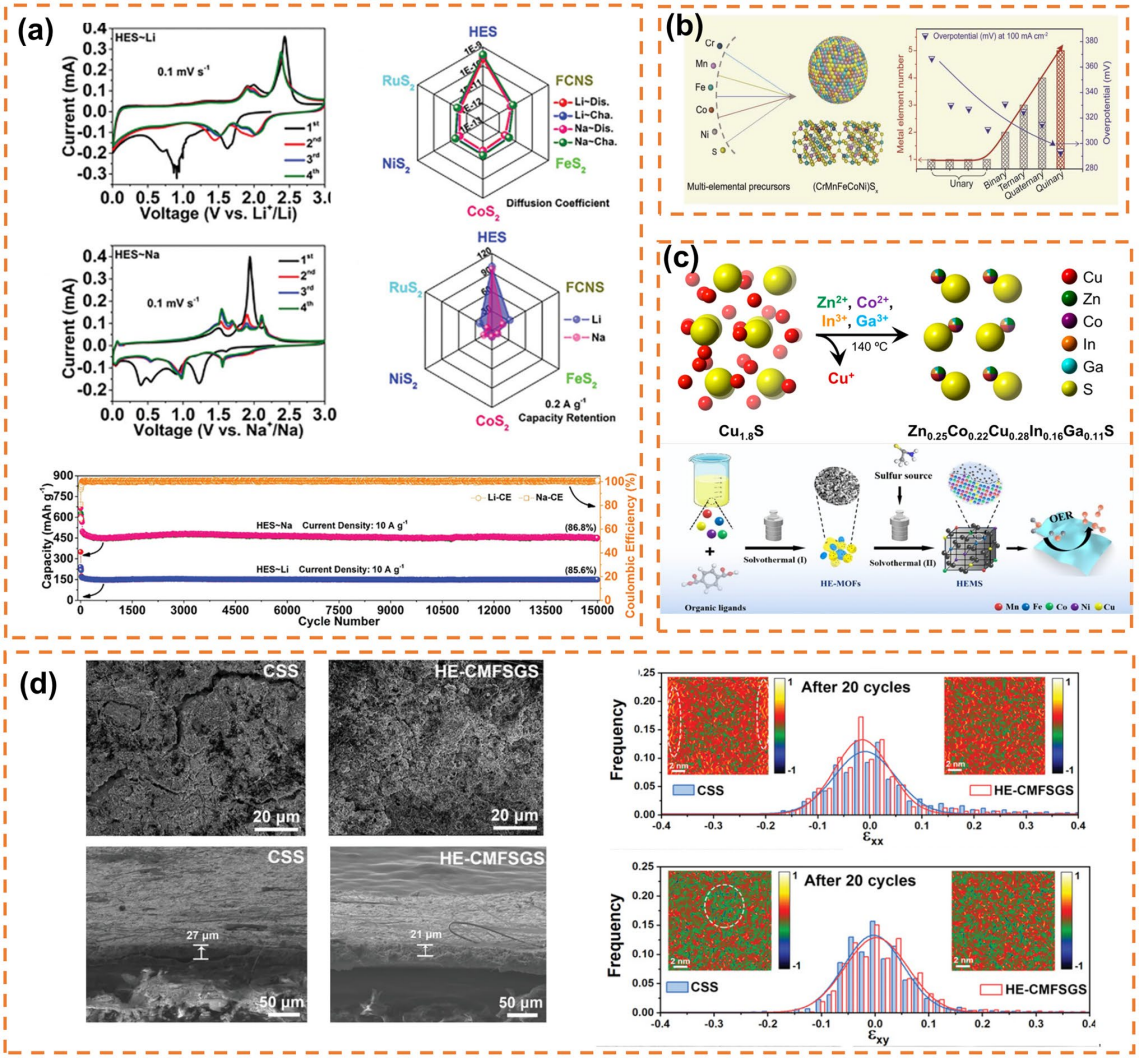
**a** CV curve of high-entropy sulfide (FeCoNiCuRu)S_2_ and (FeCoNiCuRu)S in a lithium-ion half battery and the comparison of diffusion coefficients of lithium and cyclic stability. Reprinted with permission from Ref. [165]. Copyright 2023, Wiley‐VCH GmbH.** b** Structure diagram of high-entropy nanoparticle (CrMnFeCoNi)S_x_ and schematic diagram of its OER catalysis. Reprinted with permission from Ref. [161]. Copyright 2020, Wiley‐VCH GmbH.** c** Synthesis diagram of high-entropy molten zinc metal sulfide (Zn_0.25_Co_0.22_Cu_0.28_In_0.16_Ga_0.11_)S. Reprinted with permission from Ref. [166]. Copyright 2021, American Chemical Society. **d** SEM image of the CSS electrode and HE-CMFSGCS electrode, as well as their cross-sections after 20 cycles and TEM-HAADF images of the pole plates of the CSS electrode and HE-CMFSGCS electrode after 20 cycles. Reprinted with permission from Ref. [167]. Copyright 2022, Wiley‐VCH GmbH

Solvent thermal method of sulfide is generally by putting the synthetic precursor (high-entropy MOF, etc.) and sulfur source (thioacetamide, etc.) into a hydrothermal kettle under heating and pressure in proportion. This synthetic method is simple and the conditions are relatively mild, which is favored by the majority of researchers and is one of the commonly used means of synthesis of high-entropy sulfides. Li et al. [157] prepared high-entropy sulfide (MnFeCoNiCu)S_2_ by two-step solvothermal method with MOFs as precursor and verified its catalytic activity for OER ect., proposed 7 HES with Pnma structure (M:S≈1:1) and 3 HES with Pa-3 structure (M:S = 1:2), and found that the addition of Mo can improve the catalytic performance [158]. Nguyen et al. [159] prepared sulfate high-entropy sulfide FeNiCoCrXS_2_ (*X* = Mn, Cu, Zn, or Al) by two-step solvothermal method and verified its excellent OER activity. Xu et al. [160] prepared high-entropy sulfide (CdZnCuCoFe)S_x_ by one-step solvothermal method and found that it can selectively photocatalytically produce CO from biomass polysaccharides and peroxides. Cui et al. [161] first synthesized high-entropy metal sulfide CrMnFeCoNi)S_x_ by solvothermal method with good OER catalytic activity (Fig. 6b). Liao et al. obtained high-entropy metal disulfide nanospheres by solvothermal method from high-entropy glycerol spherical sulfuration [152]. Wang et al. [162] used one-step solvothermal method to grow HES-FeCoNiCrMnS_2_ in situ on carbon cloth as electrode for assembling mixed acid–base glycerol fuel cell (AA-DGFC), showing excellent stability. For high-entropy selenite, current research mainly focuses on its application in the field of catalysis. Yao et al. prepared high-entropy selenite (CoNiCuMnMo)Se by simple hydrothermal method and found that it has good catalytic effect (1.20 V at 10 mA cm^−2^) and stability in glycerol oxidation reaction (GOR) [163]. Jiang et al. prepared flower-like high-entropy selenite (CoNiFeCuCr)Se (F-HES) by two-step solvothermal method and showed excellent OER activity (252 mV at 100 mA cm^−2^) and stability (50 h) [164].

#### *Solid-State* Reaction Method

The solid-state reaction method of sulfides is generally in an inert atmosphere, after a variety of metal sulfides and sulfur powder are fully mixed by ball milling or grinding, and then annealed at high temperatures, usually above 500 °C [153]. Deng et al. synthesized high-entropy chromium alloy polycrystal ingot with composition of (GeSnPb)_1/3_ (SSeTe)_1/3_ and Bi or Na doped samples by two-step solid-state reaction process, and used the disorder of anionic and cationic sublattices to prove the stability of single-phase solid solution in rock salt crystal structure [74]. Cavin et al. predicted and synthesized two-dimensional high-entropy transition metal sulfide (MoWVNbTa)S_2_ by calcination annealing method for the catalytic conversion of CO_2_ to CO [168]. Transition metal sulfides have excellent performance as sodium storage negative electrode materials due to their rich redox sites and good electronic conductivity. However, due to the repeated sodium/denaturation process, the structure degradation and volume expansion effect lead to poor cycling performance of the material, limiting the applicability of the material. Cheng et al. [165] prepared pressure-stable (FeCoNiCuRu)S_2_ by grinding and calcining the raw materials, which has long-term stability and maintains 92% retention rate after 15,000 cycles at 5 A g^−1^ (Fig. 6a). Chien et al. [169] proposed the concept of high entropy in bismuth metal phosphorus trisulfide (MPS3) as anode material for potassium ions, prepared MPS3 by traditional solid-phase reaction and found that the high-entropy materials would undergo electrochemical recombination during cycling, resulting in alloy precipitation and formation of flaky structure, enhancing mechanical stability and reducing mechanical stress during K ion insertion/extraction. Chang et al. [170] prepared high-entropy rock salt sulfide AgSnSbSe_1.5_Te_1.5_ by one-step melting method and proved that various heterogeneous interfaces and metal nanoparticles with different functions were formed due to the participation of active and inactive metals in the phase transformation reaction, reducing the diffusion energy barrier of K^+^ and inhibiting potential shuttle effect.

#### Cation Exchange Method

In 2020, Benjamin C. Steimle and his colleagues developed a method to synthesize scalable nanoparticles by modifying various types of nanoparticles through cation exchange reactions. In these reactions, the cations in sulfides, selenides, and other nanoparticles are substituted by the cations present in the solution, resulting in nanostructures with heterogenous features containing multiple material phases [154]. This technique is commonly known as cation exchange. The process involves utilizing a model system based on metal sulfide, incorporating a solvent, stabilizing ligands, and a Lewis base as a driving force. Subsequently, multiple sequential exchange solutions (comprising other metal salts) are introduced to facilitate cation exchange reactions and fabricate heterostructures of sulfides with multiple metal components. Even though the conditions throughout the entire procedure are mild, the selection of suitable model systems is essential. Connor R. McCormick extended the cation exchange approach and fabricated Zn_0.25_Co_0.22_Cu_0.28_In_0.16_Ga_0.11_S nanoparticles (Fig. 6c) in colloidal form [166]. Yuanting Lei and collaborators adopted a gentle cation exchange method to synthesize a novel high-entropy Co–Zn–Cd–Cu–Mn sulfide (CoZnCdCuMnS@CF) nanoarray supported on carbon fibers, which demonstrated exceptional durability in catalyzing both the HER and the OER [171].

#### Mechanical Alloying Method

The mechanical alloying method of sulfide is a technique for obtaining polymetallic sulfides by subjecting various metal sulfides and pure sulfur to high-energy ball milling in a ball milling tank. This approach eliminates the need for external heating, relying solely on the heat generated through friction during ball milling to provide energy. Although the conditions are relatively mild, the corresponding reaction time is longer, typically exceeding 60 h, and it requires prior synthesis of single metal sulfide precursors [74, 155, 172]. Zhao et al. employed the high-energy ball milling method to synthesize Cu_4_MnFeSnGeS_8_ anodes, which enhanced both reversible crystal phase transformation and mechanical stability (Fig. 6d), thereby improving cycling stability. In the sodium-ion battery, after 200 charge and discharge cycles, the reversible capacity of 569.2 mA h^−1^ can still be maintained, and the capacity retention rate of the battery is close to 100% [167]. High-entropy rock salt sulfide (HEMC) exhibits promising prospects for development in potassium-ion batteries (PIB).

In conclusion, the advantages and disadvantages of the solid-state reaction method, solid-phase reaction method, and mechanical alloying method for high-entropy sulfides are similar to those for high-entropy oxides. However, a key difference is that the solid-state reaction method for sulfides must be conducted in an inert atmosphere to prevent the incorporation of oxygen. As a result, the conditions are more stringent compared to those for oxides. On the other hand, the cation exchange method offers milder synthesis conditions and allows for gradual increases in the types of metal elements. Nonetheless, this approach involves complex steps and has historically limited choices in metal elements.

### High-Entropy Carbides/Nitrides

The traditional method for the synthesis of carbides is carbothermal reduction with the reaction equation:

4 $${\text{MeO}}_{\text{X}}+\text{C}\to \text{MeC}+\text{CO}(\text{g})$$

**Fig. 7:**
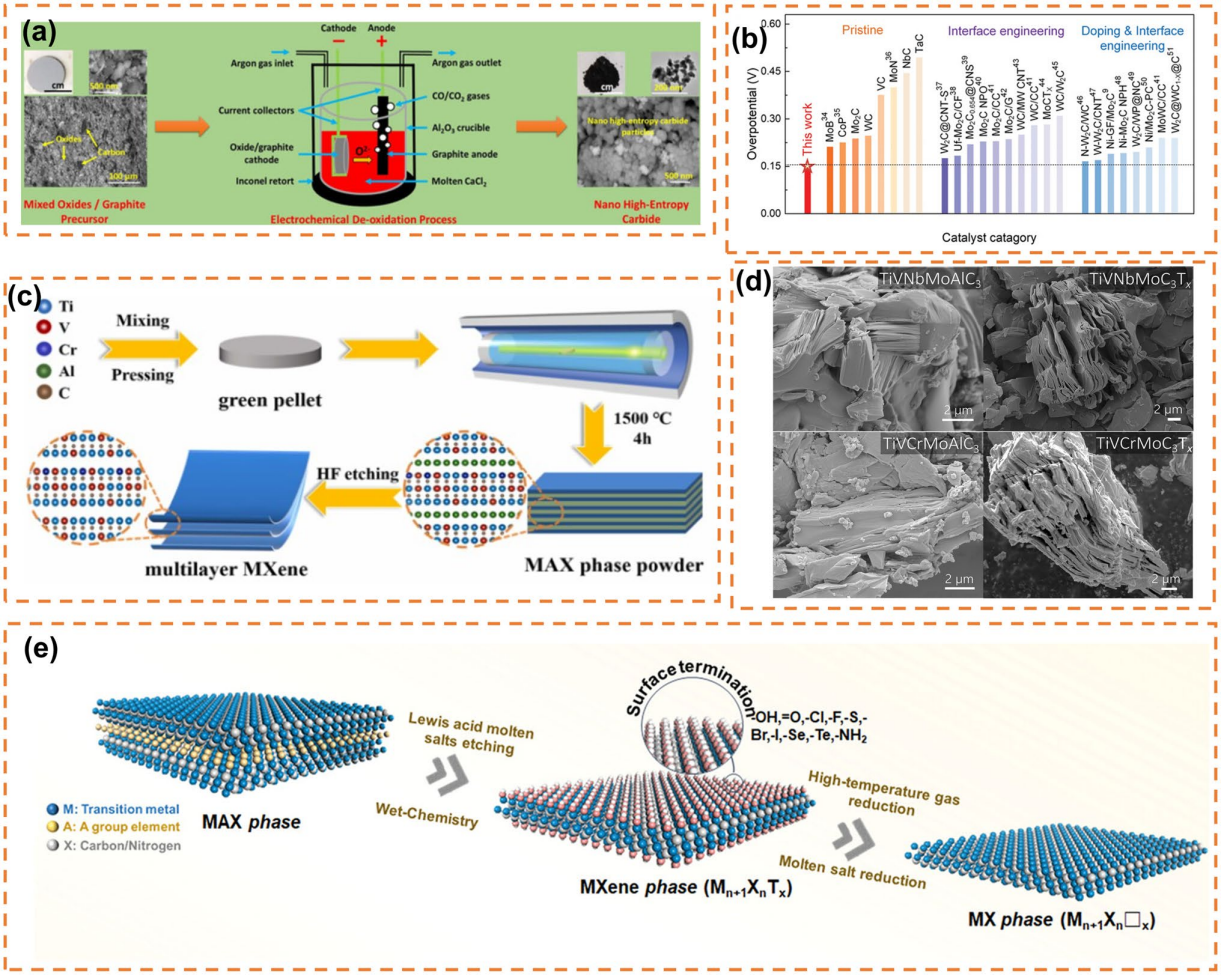
**a** A simple electrochemical method for the synthesis of (TiNbT_a_ZrHf)C. Reprinted with permission from Ref. [177]. Copyright 2020, Wiley‐VCH Verlag GmbH & Co. KGaA.** b** Comparison of η_10_ between the HECNPs and other nonprecious-metal-based catalysts. Reprinted with permission from Ref. [179]. Copyright 2022, Wiley‐VCH GmbH. **c** Schematic illustration of the fabrication of MXenes. Reprinted with permission from Ref. [180]. Copyright 2022, Elsevier Ltd. **d** SEM micrographs of high-entropy MAX and MXenes. Reprinted with permission from Ref. [75]. Copyright 2021, American Chemical Society.** e** Structure diagram of MAX to MXene and then to MX. Reprinted with permission from Ref. [181]. Copyright 2023, Wiley‐VCH GmbH

However, this process can only react positively at very high temperatures, with high energy consumption, in addition to the tendency to produce coarser micron-sized particles at high temperatures, which do not satisfy the requirements of catalysis and greatly limit the application of carbons [173]. In 2000, researchers were dedicated to discovering an alternative method to replace the conventional carbothermal reduction process. Numerous molten salt-based electrochemical methods have been identified, which can dissolve oxide anions and transport them to the anode for discharge, effectively reducing the kinetic barrier of breaking metal–oxygen bonds. However, these methods also suffer from increased side reactions [174, 175]. In 2019, Li et al. [176] prepared V_2_(A_*x*_Sn_1−*x*_)C (A = Fe, Co, Ni, Mn) high-entropy MAX phase carbides by mixing and grinding a certain proportion of V, Sn, C, Fe, Co, Ni, Mn powders with NaCl and KCl and heating them to 1100 °C in a tubular furnace, and controlled their magnetism by adjusting the combination of A. Sure et al. [177] first synthesized the ultra-high-temperature high-entropy carbides (TiNbTaZrHf)C (Fig. 7a) by a simple electrochemical method, and found that it has good cycling performance in supercapacitors (87% capacity retention after 2000 complete cycles). Yang et al. [178] prepared (VNbTaZrHf)C high-entropy carbides nanoparticles by electrochemical method, and found that it achieved 50 F g^−1^ specific capacitance in 1 M KOH at a scanning rate of 10–100 mV s^−1^ in supercapacitors, with a capacity retention rate of 89% after 2500 complete cycles, showing excellent capacitance performance. Zhang et al. synthesized porous carbide powders (FeCoCrMnNi)C by a solgel method and found that the best capacitance performance was achieved when the carbon source (glucose) was added at 5 g during the synthesis process (specific capacity reached 169.7 F g^−1^ with a current density of 0.5 A g^−1^).

The diverse physicochemical properties of carbides in morphology, composition, and microstructure contribute significantly to their applications in catalysis and energy storage. Harrington et al. investigated the phase formation of twelve different five-metal high-entropy carbides and observed that the addition of tungsten and molybdenum to the IVB or VB transition metal system decreased the likelihood of single-phase formation; however, they were able to synthesize a system with Mo and W single phases. The stability of these phases is determined by a trade-off between enthalpy and entropy [182]. Niu et al. [179] obtained defective 10-nm high-entropy (MoWVNbTa)C nanoparticles through centrifugation of waste liquid from wire-cut electrical discharge machining of high-entropy carbides, which exhibited excellent catalytic activity and stability for the HER reaction (Fig. 7b).

#### Two-Dimensional Transition Metal Carbon (Nitrogen) Compounds

High-entropy two-dimensional transition metal carbamates (MXenes) are typically obtained through chemical etching to eliminate the A layer from high-entropy three-dimensional layered carbamates (MAX). The synthesis of MAX precursors can be achieved via carbothermal reduction or molten salt electrochemistry, and the resulting MXene powders are collected by stirring followed by washing in hydrofluoric acid. Ma et al. successfully synthesized high-entropy carbamate Ti_2_V_0.9_Cr_0.1_C_2_T_*x*_ MXenes (Fig. 7c) using this method and observed excellent capacitance performance (553.27 F g^−1^ at 2 mV s^−1^) [180]. Nemani et al., on the other hand, synthesized two high-entropy MAX compounds, TiVNbMoAlC_3_ and TiVCrMoAlC_3_, which were subsequently converted into high-entropy TiVNbMoC_3_T_*x*_ and TiVCrMoC_3_T_*x*_ MXenes with equal molar ratios (Fig. 7d); their findings confirmed the feasibility of synthesizing additional high-entropy MXenes through experimental and computational approaches [75]. Zhou et al., meanwhile, obtained Ti_1.1_V_0.7_Cr_*x*_Nb_1.0_Ta_0.6_C_3_T_*z*_ MXene monoliths via etching of (Ti_0.8_V_0.8_Cr_0.8_Nb_0.8_Ta_0.8_)AlC_3_ MAX precursor material, demonstrating a remarkable volume capacitance of up to 1688 F cm^−3^ (490 F g^−1^ at 2 mV s^−1^) [183].

MXenes compositions have also been utilized in lithium-ion batteries owing to their unique two-dimensional structure and the synergistic effect between polymetallic ions. Etman et al. synthesized a high-entropy MXene, Ti_1.1_V_0.7_Cr_*x*_Nb_1.0_Ta_0.6_C_3_T_*z*_ (T_*z*_ = –F, –O, –OH), through solid-phase reaction as the negative electrode material for lithium-ion batteries, exhibiting a capacity of 126 mAh g^−1^ at 0.01 A g^−1^ [184]. Wu et al., on the other hand, employed MXenes in lithium-sulfur batteries and summarized the catalytic functions of MXene and MXene-based heterostructures in sulfur cathodes and lithium anodes respectively [185]. Du et al. [186] obtained a high-entropy carbon nitride MAX phase Ti_1/3_V_1/6_Zr_1/6_Nb_1/6_Ta_1/6_)_2_AlC_x_N_(1-x)_ by metallization of medium entropy nitride MAX (Zr_1/3_Nb_1/3_Ta_1/3_)_2_AlC, Ti_4_AlN_3_ and V_2_AlC; they discovered that incorporating medium entropy MAX phase with configuration entropy of 1.1R during synthesis process prevented phase separation of high-entropy nitrides successfully. After etching Ti_1/3_V_1/6_Zr_1/6_Nb_1/6_Ta_1/6_)_2_AlC_x_N_(1-x)_, they observed excellent electrochemical performance of high-entropy nitride MXenes in lithium-sulfur batteries (863 mAh g^−1^ at 0.5C after 50 cycles).

In addition to the conventional etching methods of MXenes, such as molten salt electrochemical method and hydrothermal method, further research has explored their application in MAX phase etching to obtain MXenes. These methods introduce surface groups like –Cl, –O, and –OH into MXenes (Fig. 7e), which subsequently influence the performance of MXene materials [187]. However, subjecting MXenes to high-temperature treatment in a hydrogen environment effectively eliminates most of these surface functional groups, resulting in the formation of pristine MXene (MX). Distinguished from its parent compounds, MX exhibits a novel electronic structure and a unique set of catalytic activity centers that offer significant advantages over traditional precious metals in terms of catalytic efficiency, selectivity, and activity [181].

### High-Entropy Metal–Organic Frameworks

The metal–organic framework (MOF) is a porous coordination polymer with controllable morphology, high specific surface area, rich pore structure, and multifunctionality. Since its initial proposal by Yaghi et al. [188] in the late 1990s, MOF materials have garnered significant interest among researchers. The solvent heating method stands as the primary synthetic approach for MOFs due to its simplicity, rapid reaction kinetics, and mild conditions; thus holding promising prospects for further development in MOF synthesis. In addition, the synthetic methods of MOF include electrodeposition [189], microwave synthesis [190], mechanochemical synthesis [191], and spray drying synthesis [192], but the stable entropy-driven mechanism of high-entropy MOFs, their practical operation often leads to decomposition and generation of various derivatives; hence they find applications primarily in hydrogen evolution [193], oxygen evolution [194], and N_2_ fixation processes [195]. Xinhui Zhao [193] first proposed the concept of high-entropy MOF in 2019, synthesized HE-MOF containing Mn, Fe, Co, Ni, Cu using solvothermal method, and verified its electrocatalytic activity for OER. Currently, the prevailing synthetic methods for high-entropy MOF include solvent thermal method [196], mechanical chemical synthesis method [197], and electrodeposition method [189]. however, there is a scarcity of relevant literature. In general, the development of synthetic strategies for high-entropy MOF is still in its nascent stage. Moreover, different characteristics can be achieved in high-entropy MOFs through coordination with diverse metals within the organic framework. Furthermore, within the field of electrochemical energy storage systems, high-entropy MOFs exhibit great potential as negative electrode materials for batteries owing to their highly adjustable ligand frameworks and coordinated effects between metals.

#### Solvothermal Method

**Fig. 8:**
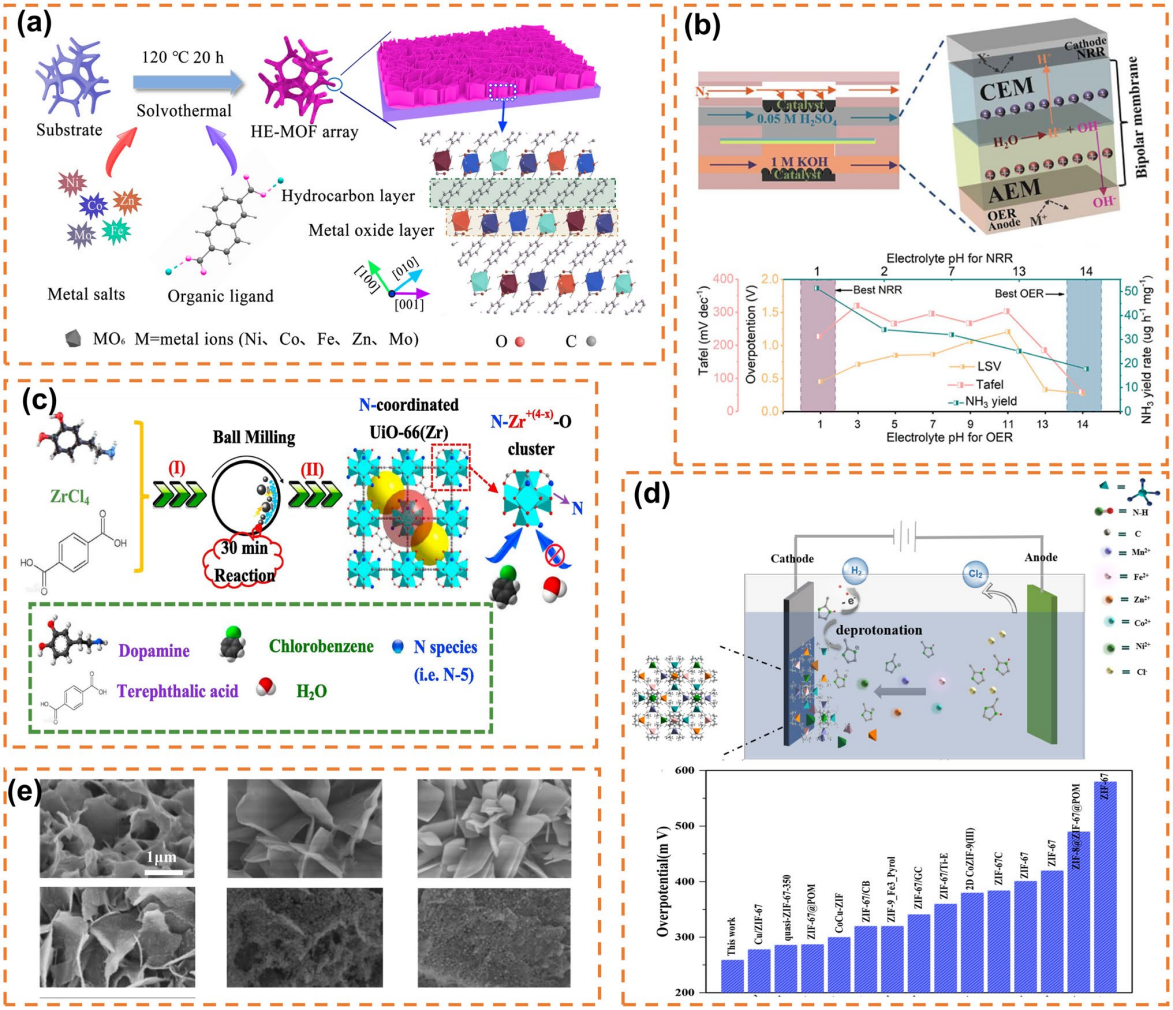
**a** Synthetic process of the 2D HE-MOF array. Reprinted with permission from Ref. [196]. Copyright 2022, American Chemical Society. **b** Electrolytic schematic of the BPM-based flow-type cell and NH_3_ yield at different pH electrolytes, overpotential at 10 mA cm^−2^. Reprinted with permission from Ref. [195]. Copyright 2024, Copyright Clearance Center, Inc. **c** Schematic illustration of N-coordinated UiO-66(Zr) material with dopamine prepared via green and fast mechanochemical method. Reprinted with permission from Ref. [197]. Copyright 2017, Elsevier B.V. **d** Schematic illustration of electrode position process of HE-ZIF/NF and Comparison of OER performances of HE-ZIF/NF-400 s with other reported electrocatalysts. Reprinted with permission from Ref. [200]. Copyright 2023, Hydrogen Energy Publications LLC. Published by Elsevier Ltd. All rights reserved. **e** SEM images of HE-MOF and its derivatives at different temperatures. Reprinted with permission from Ref. [194]. Copyright 1969, Elsevier

Solvothermal method is one of the most widely used methods for the synthesis of MOF. It is a reaction by dissolving organic ligand and metal salt solution together and adding initiator in a hydrothermal kettle under the control of temperature and pressure (Fig. 8a). In the synthesis of high-entropy MOFs, the synergistic effect of the presence of multiple metals will lead to interesting phenomena when metals and ligands are coordinated [198]. Xu et al. prepared NiCoFeZnMo high-entropy two-dimensional MOF with 2,6-naphthalenedicarboxylic acid tetrahydrate as the organic ligand by solvent-thermal method and found that it has excellent activity in the OER (overpotential of 254 mV at a current density of 50 mA cm^−2^) [196]. Jing Hu prepared MnFeCoNiCuZn porous hollow high-entropy MOF-74 with 2,5-dihydroxypentyl diacetic acid as the organic ligand by hydrothermal method and obtained high-entropy MOF-74 derivatives as electrocatalysts for ORR by annealing them at high temperatures [199]. NiCoFeZnV-based HE-MOFs and their derivatives (HE-MOF-H and HE-MOF-OH) were synthesized via solvothermal method by Sun et al. These materials were utilized as electrolytes in BPM flow batteries, where they exhibited catalytic activity toward NRR at the negative electrode and OER at the positive electrode (Fig. 8b) [195].

#### Mechanochemical Methods

The mechanical synthesis of MOF involves dissolving metal salt and organic ligand in a suitable solvent, followed by the addition of an initiator and vigorous stirring to synthesize MOF (Fig. 8c) [197]. This method is simpler and milder compared to the solvent thermal method, making it highly attractive to researchers. Li et al. successfully incorporated 6 coordination nickel clusters into the structure of ZIF-8 through one-pot mechanical synthesis, and also synthesized Ni-substituted ZIF-8 single crystals with a similar coordination environment using the solvent thermal method, which exhibited exceptional photocatalytic activity [201].

#### Electrodeposition

The electrodeposition method of MOF is a highly efficient, cost-effective, and scalable approach for synthesizing high-entropy MOFs. The electrodeposition reaction is governed by the mass transfer process of metal cations at significantly elevated potentials, thereby eliminating the coordination ability effect between different ions. Consequently, this enables effective coordination between the metal and ligand, facilitating the synthesis of high-entropy MOFs. Dong et al. successfully deposited high-entropy ZIF on a foamed nickel substrate via electrodeposition (Fig. 8d) and observed its remarkable electrocatalytic activity toward the OER [200].

#### MOF Derivatives

In addition to its excellent electrochemical and energy storage potential, high-entropy MOF can also be used as a self-sacrificing template to prepare high-entropy alloys [202], high-entropy sulfides [203], high-entropy selenite [204], etc. Zhao et al. prepared FeCoNiCuMnZn high-entropy MOF with terephthalic acid as a ligand by hydrothermal method, and prepared FeCoNiCuMn-NPs with this precursor by high-temperature annealing, and found that it had excellent OER catalytic performance (current density of 10 mA cm^−2^, overpotential of 196 mV) [202]. Li et al. [157] synthesized MnFeCoNiCu, CrMnFeNiCu, and FeCoNiCuMo high-entropy MOF with terephthalic acid as a ligand by solvothermal method, and prepared high-entropy metal sulfide nanoparticles with this template by hydrothermal method, which have potential applications as electrocatalysts for enhancing OER. Liu et al. [205] prepared MnFeCoNiCu high-entropy MOF with terephthalic acid as a ligand by hydrothermal method, and obtained high-entropy oxides with different morphologies by pyrolysis and annealing at different temperatures (Fig. 8e). It was found that the high-entropy oxides obtained by pyrolysis at 350 °C in Ar atmosphere and annealing at 200 °C in O_2_ atmosphere had good OER catalytic activity (current density of 50 mA cm^−2^, overpotential as low as 266 mV) [194].

### High-Entropy Composite Materials

The utilization of high-entropy materials has garnered significant attention from researchers; however, the inherent limitations of simple high-entropy materials necessitate further enhancements in order to optimize their performance. Consequently, researchers have commenced incorporating additional materials into conventional high-entropy materials to address these performance deficiencies.

**Fig. 9:**
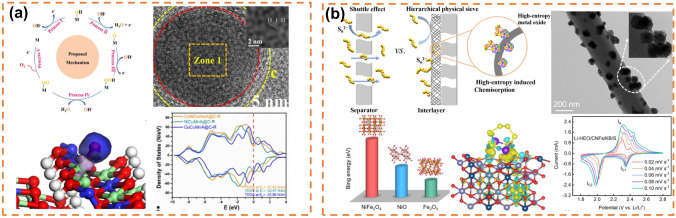
**a** Schematic diagram of an OER catalyst for carbon-clad core–shell high-entropy alloy (CoNiCuMnAl@C), along with HRTEM image and charge density difference of O adsorbed at Ni, as well as a TDOS diagram. Reprinted with permission from Ref. [209]. Copyright 2021, Elsevier B.V. **b** Diagram illustrating the dual-function effect of grapevine HEO/CNFs, along with its TEM image and comparison of binding energies and CV curves. Reprinted with permission from Ref. [211]. Copyright 2022, Elsevier B.V. on behalf of KeAi Communications Co, Ltd

Wei et al. obtained np-HEA@HEO composites by annealing and oxidizing high-entropy alloy nanoparticles and verified their excellent lithium storage capacity in the negative electrode of lithium-ion capacitors [206]. Yuan et al. prepared MF/MnO_2_ composites by doping FeCrCoMnNiAl_0.75_ high-entropy alloy in MnO_2_ by cyclic pulse electrodeposition and used them as supercapacitor electrodes, finding that they had excellent capacitance performance (961 F g^−1^ at a current density of 5 A g^−1^) [207]. Guo et al. prepared HEO@G composites by hydrothermal composite of high-entropy oxides and graphene and used them as negative electrode materials of lithium-ion batteries with excellent capacity of 950 mAh g^−1^ after 100 cycles at 200 mA g^−1^ [208]. Wang et al. prepared CoNiCuMnAl@C shell nanoparticles by pyrolysis of MOF precursors (Fig. 9a) and found their catalytic activity for alkaline OER (Fig. 9a) [209]. Jishnu et al. obtained composites by ultrasonic treatment of TiZrVCrNi high-entropy alloy nanoparticles and MoS_2_ nanoparticles and found that they could reduce the reaction of Au electrode with FLP, thereby improving the sensitivity of the response to triethylamine [210]. Fan et al. [211] calcined the nanocomposites electrospun from polyacrylonitrile and metal salts at 800 °C to obtain the composites HEO/CNFs (Fig. 9b) of high-entropy metal oxide (Cu_0.7_Ni_0.6_Fe_0.6_Sn_0.5_Mn_0.4_)O_4_ and grapevine-shaped carbon nanofibers, and found that they could improve the electrode dynamics as cathode materials for lithium-sulfur batteries, anchor LiPSs at the cathode side, significantly alleviate the shuttle effect and improve the cycling stability (Fig. 9b).

### Other High-Entropy Materials

In addition to the six types of high-entropy materials mentioned above, there are numerous other high-entropy materials, such as high-entropy diboride [212], high-entropy silicides [213], high-entropy salts, and various other high-entropy ceramic materials utilized as structural materials. These materials are primarily employed for their structural properties and exhibit high hardness, thereby enhancing the mechanical strength of electrodes. Furthermore, when rare earth elements are present in these high-entropy materials, they are referred to as high-entropy rare earth materials (HE-RE materials). This category includes HE-RE alloys, HE-RE transition metal oxides, and HE-RE carbides et al. Due to their similar atomic radii and unique electronic structures with 4*f* orbital shielding properties [214], rare earth elements often contribute superior performance characteristics to high-entropy materials. As a result, they hold potential advantages in the fields of electrocatalysis and energy storage. However, current research on the application of HE-RE materials is primarily focused on structural material applications. There is a lack of investigations into their electrocatalytic and energy storage capabilities, which remains relatively scarce in the literature. Consequently, it can be inferred that high-entropy rare earth materials represent a promising class of electrode materials for future development opportunities.

High-entropy hydroxides (HEH) are a new type of high-entropy material that also has considerable potential in the field of catalysis [215–217], but due to the instability of hydroxides at high temperatures, they easily decompose into oxides at high temperatures, which greatly restricts the synthesis of high-entropy hydroxides and can only be carried out under relatively mild conditions. The commonly used synthesis methods include solvothermal method [218–221], laser pulse method [222], and electrochemical synthesis method [217, 223]. Layered double hydroxides (LDH) are hydroxides that are stacked in layers, and high-entropy layered double hydroxides (HE-LDH) have good application effects in glucose oxidation reaction (GOR) [221] and supercapacitor positive electrode materials due to their layered structure and many active sites [224]. However, high-entropy hydroxides still face the limitations of traditional hydroxides, and their poor high-temperature stability remains a significant factor preventing further application in various fields.

High-entropy intermetallic compounds (HEI) are multimetal alloys consisting of more than five metal elements. Unlike high-entropy alloys (HEA), where the atoms are randomly distributed, the crystal structure of intermetallic compounds originates from binary or multicomponent metal precursors. Due to their higher ordered structure compared to HEA, HEI can achieve the isolation of specific atoms and has the potential for structural and scale regulation [225]. This makes it highly promising in the fields of catalysis and energy storage. The preparation methods of HEI are similar to those of alloys [226, 227]. However, more advanced synthesis methods are needed to further regulate the element composition and structure of HEI. For instance, Soliman et al. synthesized HEI colloidal particles using a low-temperature liquid-phase method and observed detailed changes in morphology, composition, and structure during the particle formation process [228]. In terms of application, HEI demonstrates excellent performance in catalyzing the dehydrogenation of propane [229], HER [225, 230], and ORR [231]. It also shows great potential in zinc-air battery applications [231].

## Conclusion and Outlook

This study examines various high-entropy electrode materials, encompassing high-entropy alloy nanoparticles, oxides, phosphorus/sulfides, carbon/nitrides, MOFs, and composite materials. It elaborates on the synthesis techniques for these materials and summarizes their performance and utilization as electrodes in electrocatalysis and energy storage applications. Additionally, it presents a concise overview of the high-entropy concept and its initial evolution, partially tackling the complexities associated with the diverse preparation methods for high-entropy electrode materials.

In terms of preparation methods, the carbon thermal shock method has been widely used for synthesizing high-entropy alloys and high-entropy carbon/nitrogen compounds due to its relatively traditional approach and fast preparation speed. However, its drawbacks are also apparent as it requires high temperature and equipment demands, which limits further development. On the other hand, solid-state reaction is a common method for synthesizing high-entropy sulfides, selenites, oxides and other compounds with simple synthesis procedures, low equipment requirements and reaction temperatures usually in hundreds of degrees Celsius. Nevertheless, exploring the reaction conditions is crucial since different proportions of precursor reactions at varying temperatures may result in nonunique synthetic material phases even at the same temperature. Solvent thermal method, low-temperature liquid-phase method, cation exchange method, solution combustion method and electrodeposition method are milder alternatives that are easy to operate with green and clean features suitable for laboratory environments but not ideal for large-scale production due to container size limitations. It is worth mentioning that using MOF as a precursor has become a hot topic in recent years for preparing high-entropy materials because this approach offers great adjustability where microstructure can be controlled by changing ligands while providing diverse metal element choices with huge potential for development.

In terms of performance application, high-entropy electrode materials are primarily utilized in electrocatalysis and energy storage applications. They do not prioritize high mechanical strength but instead focus on the morphology, phase state, entropy value, and types of transition metals. High-entropy alloys typically manifest as nanoparticles and nanowires in the field of electrocatalysis, leveraging their nanoscale microstructure and diverse metal element characteristics to catalyze hydrogen evolution and oxygen evolution reactions. However, due to their alloy nature, they experience numerous side reactions in the electrolyte, resulting in a relatively slow adoption for energy storage purposes. Nevertheless, they have also been reported as separators for lithium-sulfur batteries. High-entropy oxides exhibit rich structures and find extensive use in electrocatalysis and energy storage owing to the involvement of oxygen elements. Expanding the range of elements not only increases the entropy value but also alters material phase states. Different oxide structures are employed across various fields with incomplete phase states; for instance, spinel and rock salt oxides are commonly used in lithium-ion batteries as well as oxygen evolution/hydrogen evolution catalysis while perovskite oxides find application in thermoelectric fields. Although less explored than oxides, high-entropy chalcogenide compounds possess significant potential with excellent performance observed in thermoelectricity generation systems or electrocatalytic processes such as lithium-sulfur batteries or sodium batteries. High-entropy MXene within high-entropy carbonaceous compounds exhibits a unique two-dimensional structure along with synergistic effects between polymetallic components that make it highly suitable for electrode materials used in electrocatalysis and energy storage applications; however, research on high-entropy MXene remains limited despite its promising future prospects.

Despite the excellent performance of high-entropy materials, further development in this field requires concerted efforts from all directions.

1. Due to the complex composition of various elements in high-entropy materials, there is immiscibility among the components, preventing the formation of a single phase. This seriously restricts the synthesis of high-entropy materials and hinders their ability to exhibit all their characteristic properties. Existing synthesis methods primarily rely on high temperature and high pressure means to address these challenges. Therefore, it is crucial to explore low-temperature synthesis methods for high-entropy materials that can accommodate a wide range of elements.
2. Previous studies have extensively explored the morphology of high-entropy materials, with a focus on reducing their scale to the nanometer level and preparing nanoparticles with regular morphology. However, due to the lattice distortion effect of high-entropy materials, controlling the morphology is not as straightforward as it is for single-phase materials. Therefore, it is crucial to investigate the relationship between composition and morphology in order to prepare high-entropy materials with adjustable morphologies.
3. The synthesis of high-entropy materials often necessitates the use of a variety of high-purity metal elements, resulting in high costs and typically high energy consumption during large-scale preparation processes. Therefore, it is crucial to explore more cost-effective methods for preparing high-entropy materials. For instance, one approach could involve the adjustment and reconstruction of natural mineral materials to directly transform them into high-entropy electrode materials, thereby significantly reducing costs. Another possibility is to modify the elements of recycled waste electrode material in order to fully leverage the synergistic effects between the elements of the high-entropy material, ultimately achieving performance recovery or even surpassing previous levels.
4. Theoretical calculations can serve as a valuable tool for assessing the catalytic and energy storage performance of materials, as well as providing guidance for the synthesis of high-entropy materials. While previous studies have explored theoretical calculations for the synthesis of high-entropy alloys, there have been limited reports on related theoretical calculations for other high-entropy materials, such as high-entropy oxides and sulfides. Therefore, further expansion of theoretical calculation models and methods for the synthesis of high-entropy materials will be a breakthrough in advancing the development of high-entropy materials.
